# Effects of the Digital App “Support, Monitoring and Reminder Technology for Mild Dementia” (SMART4MD) on People With Mild Cognitive Impairment and Their Informal Caregivers: 18-Month Multicenter Pragmatic Randomized Controlled Trial

**DOI:** 10.2196/83123

**Published:** 2026-07-24

**Authors:** Zartashia Ghani, Poe Eindra Thant, Sanjib Saha, Peter Anderberg, Maria Quintana Aparicio, Pilar Barnestein-Fonseca, Selim Cellek, Fermín Mayoral Cleries, Maite Garolera, Gloria Guerrero-Pertiñez, Karen Hayden, Carmel Moore, Jufen Zhang, Johan Sanmartin Berglund, Johan Jarl

**Affiliations:** 1 Department of Health Blekinge Institute of Technology Karlskrona Sweden; 2 Health Economics Unit Lund University Lund Sweden; 3 Brain, Cognition and Behavior, Clinical Research Group Consorcio Sanitario de Terrassa Barcelona Spain; 4 Research Unit Instituto CUDECA de Estudios e Investigación en Cuidados Paliativos Fundación CUDECA Málaga Spain; 5 Medical Technology Research Centre Anglia Ruskin University Peterborough United Kingdom; 6 Unidad de Gestión Clínica de Salud Mental, Hospital Regional Universitario de Málaga Málaga Spain; 7 Grupo C-03: Investigación Básica, Clínica y Epidemiológica en Salud Mental, IBIMA Plataforma BIONAND, Instituto de Investigación Biomédica de Málaga Málaga Spain; 8 Anglia Ruskin Clinical Trials Unit, School of Medicine Faculty of Health, Education Medicine and Social Care Anglia Ruskin University Chelmsford United Kingdom

**Keywords:** caregiver, effectiveness, older adults, memory, mHealth, mild cognitive impairment, mild dementia, mobile app, smartphone

## Abstract

**Background:**

Previous research has shown that mobile health (mHealth) interventions are effective in reminding older adults with chronic conditions about health care appointments and promoting adherence to medication schedules. However, the evidence is limited by the short duration and poor quality of the interventions.

**Objective:**

We evaluated the effectiveness of the Support, Monitoring and Reminder Technology for Mild Dementia (SMART4MD) tablet app in improving the quality of life (QoL) of people with mild cognitive impairment (PwMCI) and their caregivers through medication reminders and health care appointment alerts.

**Methods:**

An 18-month pragmatic randomized controlled trial was conducted in Spain and Sweden from December 2017 to September 2020 and included 1078 PwMCI and their informal caregivers.

**Results:**

For PwMCI, the intervention improved the primary outcome, composite Quality of Life in Alzheimer’s Disease (QoL-AD) scale, at 18 months (mean difference 0.75, 95% CI 0.07-1.42; *P*=.03), and a similar effect was observed at 6 months (mean difference 0.73, 95% CI 0.09-1.36; *P*=.02). Confirmatory secondary outcomes for PwMCI, including medication adherence (*P*=.12) and Mini-Mental State Examination (MMSE) score (*P*=.45), did not differ significantly between groups at the 18-month follow-up. Exploratory analyses showed higher total accumulated quality-adjusted life years (QALYs) at 6 months for PwMCI (mean difference 0.035, 95% CI 0.02-0.05; nominal *P*<.001), informal caregivers (mean difference 0.050, 95% CI 0.04-0.07; nominal *P*<.001), and dyads (mean difference 0.085, 95% CI 0.06-0.11; nominal *P*<.001). A dropout rate of approximately 40% (429/1083, 39.61%) was observed in both the intervention and control groups.

**Conclusions:**

The SMART4MD intervention was associated with a modest improvement in PwMCI’s QoL as measured by the composite QoL-AD score. Evidence for benefits among informal caregivers and dyads was less consistent and should be interpreted as exploratory. Nominally significant findings for exploratory outcomes should be interpreted with caution, as they were not adjusted for multiplicity.

**Trial Registration:**

ClinicalTrials.gov NCT03325699; https://clinicaltrials.gov/ct2/show/NCT03325699

**International Registered Report Identifier (IRRID):**

RR2-10.2196/13711

## Introduction

Dementia is a syndrome characterized by progressive deterioration in several cognitive domains that interferes with activities of daily living. Dementia imposes a high health and social care burden, with an estimated global cost of US $1 trillion per year [1]. In 2020, more than 55 million people worldwide were living with dementia, a number expected to increase to 78 million by 2030 [2].

Mild cognitive impairment (MCI) is an early phase of memory loss or decline in cognitive abilities, such as visual/spatial or language skills. People with MCI (PwMCI) are at higher risk of developing dementia [3,4], with an estimated annual conversion rate of 10%-15%. In MCI, the individual’s capacity to carry out daily activities generally remains unimpaired [5]. However, activities that require higher cognitive abilities, such as telephone use, driving, shopping, cooking, and medication management, are often affected [6]. Consequently, the cognitive, behavioral, and functional symptoms of MCI negatively affect the well-being and quality of life (QoL) of PwMCI [6-8].

As cognitive impairment worsens, informal caregivers, often a spouse or child, experience a greater caregiving burden [9]. As PwMCI cope with reduced autonomy due to MCI, the role of the informal caregiver becomes essential in providing assistance and support. One crucial aspect is daily medication management, as cognitive impairment makes it challenging for PwMCI to manage their medications [10,11], which may lead to suboptimal pharmacological management, unnecessary hospitalization, increased costs and burden of illness, and premature death [12]. Thus, informal caregivers bear significant responsibility for caring for PwMCI and assisting with medication administration [11,13]. While caregiving for a family member can be fulfilling [14], informal caregivers often experience increased emotional strain and a higher risk of depressive symptoms [13,15]. Previous research has shown that caregivers of PwMCI experience twice the caregiving burden of caregivers of cognitively healthy individuals. Therefore, cognitive impairment and reduced autonomy in daily activities negatively affect the QoL of both PwMCI and their informal caregivers [13,16].

It may be feasible to reduce the burden of informal caregiving by implementing interventions that help PwMCI maintain independence in daily activities [15]. In the absence of a cure for dementia, current public health policy focuses on helping individuals live well with dementia and slowing disease progression [17]. A well-designed intervention could help PwMCI and their caregivers acquire strategies to sustain daily activities, reduce the burden associated with progressive cognitive decline, and consequently enhance overall well-being over an extended period.

Technologies such as telehealth, remote monitoring, and digital reminders can assist PwMCI. Mobile health (mHealth) interventions are effective in reminding older adults to attend health care appointments and adhere to medication schedules [18]. Additionally, mHealth interventions have been shown to improve cognitive function [19]. Smartphone and tablet apps may therefore provide a unique opportunity to support self-care among PwMCI. Introducing these supportive apps early in the cognitive impairment process may encourage continued use as the disease progresses.

The Support, Monitoring and Reminder Technology for Mild Dementia (SMART4MD) intervention aimed to achieve these goals (ClinicalTrials.gov: NCT03325699). SMART4MD was a multicenter pragmatic randomized controlled trial (RCT) conducted in Sweden, Spain, and Belgium [20]. In this study, we evaluated the effectiveness of the SMART4MD app combined with standard care compared with standard care alone.

The primary hypothesis is that the SMART4MD app, in addition to standard care, improves QoL in PwMCI. Secondary confirmatory hypotheses are that the intervention enhances medication adherence, global cognitive function, and health-related QoL in PwMCI. Furthermore, SMART4MD is hypothesized to reduce caregiver burden among informal caregivers.

## Methods

### Study Design and Setting

The SMART4MD trial targeted PwMCI and their primary informal caregivers. SMART4MD is a tablet-based health app developed by Healthbit Ltd [21] specifically for people with MCI or mild dementia and their informal caregivers. The interface design prioritizes simplicity, familiarity, and customization to reduce cognitive load. It is a tablet-first app designed for tablet screens with large interactive zones and simple line charts rather than complex data visualizations for monitoring health progress. Key features of the SMART4MD app are medication reminders, reminders for appointments with health care professionals, and cognitive training through stimulating games and photographs. The app also includes an optional feature that allows users to share information with family members regarding their daily health condition, specific health issues, and QoL [20]. The project strictly followed a user-centered design approach involving testing with dyads (PwMCI and their caregivers) in Spain, Sweden, and other partner countries [22]. The interface was designed for simultaneous use by 2 types of users: PwMCI (the primary users) and caregivers (the secondary users). The design functions as an external memory aid by supporting reminders and daily schedules, thereby reducing the mental effort required to remember daily routines. Features such as calendars and photographs mimic real-world tools, making the app familiar and intuitive for users who are less familiar with technology. The app also allows users to customize features according to personal preferences, thereby enhancing accessibility and adaptability for PwMCI.

In this study, we used data collected from 3 sites in Sweden and Spain: (1) Blekinge Institute of Technology (BTH), Karlskrona, Sweden; (2) Consorci Sanitari de Terrassa (CST), Barcelona, Spain; and (3) Servicio Andaluz de Salud (SAS), Málaga, Spain. The site in Belgium was excluded because of insufficient participation (5 dyads) and incomplete data. Health outcomes were assessed at baseline and at 6, 12, and 18 months. Additional information on the intervention is available in the trial protocol [20]. The SMART4MD trial is reported in accordance with the CONSORT (Consolidated Standards of Reporting Trials) guidelines. The CONSORT checklist is provided in Multimedia Appendix 1.

### Deviation From the Original Trial Registration

The Czech Republic was not included in this sample because the Czech study withdrew from the project due to financial constraints. As no participants were recruited from this site, it was not included in the published protocol [20] or in this study.

Regarding the inclusion criteria, the originally specified Mini-Mental State Examination (MMSE) range of 20-26 points was later expanded to 20-28 points. This adjustment was informed by O’Bryant et al [23], who demonstrated that an MMSE score of 28 provides optimal sensitivity and specificity for detecting mild dementia in individuals with self-reported memory complaints. As the revised MMSE range of 20-28 points was incorporated into the SMART4MD trial protocol, this study is consistent with the published protocol [20].

### Ethics Approval

The study was conducted in compliance with the ethical principles outlined in the Declaration of Helsinki [24]. The trial was approved by the regional ethical review board in Sweden (LU Numbers 650-00 and 744-00) and the local ethics committee in Spain (number 02-16-107-029). Dyads provided written informed consent before participation. No financial reimbursement was provided for participation in the trial. Due to the gradual deterioration in cognitive capacity among PwMCI, the study team consistently obtained renewed consent for data collection throughout the trial [20].

### Recruitment and Eligibility Screening

Participants were identified through primary care, secondary care services (memory clinics), outpatient clinics, day hospitals, specialist mental health care units, geriatric medicine units, and neurology service units. Clinical databases, such as the Swedish National Study on Aging and Care and the CST network in Spain, were screened for individuals meeting the basic age and cognitive criteria. Study staff or licensed medical providers identified individuals who had experienced cognitive impairment for more than 6 months. Potential participants were given or sent an information package containing a participant information sheet and a standard consent form. According to the standard operating protocol, there was a 24-hour cool-off period between the time a participant agreed to join the study and the meeting at which formal consent was obtained. Participant inclusion began in December 2017 and was expected to end in February 2019. Individuals aged 55 years or older who scored 20-28 on the MMSE were eligible for inclusion [25]. Additional inclusion criteria were that participants were able to manage their medications, were recipients of home care services, had no functional disability that could prevent them from using the SMART4MD app (eg, visual, hearing, or motor impairments), had an actively involved informal caregiver, and had experienced a noticeable decline in cognitive abilities for more than 6 months before inclusion.

Individuals were ineligible to participate if they scored higher than 11 on the Geriatric Depression Scale [26] or had a terminal illness with a life expectancy of less than 3 years, as determined by the research nurse during the initial visit. Furthermore, participants were excluded if they had any other known conditions that could affect cognitive impairment, such as substance abuse or psychiatric disorders including schizophrenia, bipolar disorder, and developmental disorders.

### Sample Size Determination

The sample size was determined based on the primary outcome measure, QoL, assessed using the Quality of Life in Alzheimer’s Disease (QoL-AD) scale for PwMCI. The protocol defined a recruitment target of 1200 PwMCI-caregiver dyads, split equally between the intervention and control groups. To detect a small effect size (0.2) between the control and intervention groups with 80% power using a 2-sided Student *t* test at a 5% significance level, a minimum of 394 participants per group (788 in total) was required. Assuming a 20% dropout rate, the required sample size increased to 493 participants per group (986 in total). The assumed minimum detectable effect size of 0.2 refers to a standardized mean difference, that is, 0.2 SD units on the QoL-AD outcome, corresponding to a small effect size. As the primary outcome for the sample size calculation was the PwMCI QoL-AD score, no separate adjustment was made for within-dyad correlation in the power calculation. Furthermore, the sample size calculation did not include an explicit inflation factor for site-level clustering or correlation; the estimate was based on a simple 2-group comparison rather than a cluster-adjusted design effect.

The SMART4MD trial successfully enrolled 1083 dyads consisting of PwMCI and their primary informal caregivers, which exceeded the minimum required sample size based on the power calculation. Twenty-six dyads were excluded from the analysis because the primary informal caregiver changed during the trial.

### Intervention and Control Group

The duration of the trial was 18 months. Dyads were randomized, based on predetermined sequences, to either the intervention group (n=537) or the control group (n=541) in blocks using a 1:1 ratio through an internet-based randomization system (ALEA; FormsVision BV) established by the Anglia Ruskin Clinical Trials Unit. Dyads, research nurses, and researchers were not blinded to group assignments because of the nature of the intervention. Participants in the intervention group received standard care and the SMART4MD app on tablets for daily use by PwMCI. All dyads in the intervention group underwent a 1.5-hour training session on app use conducted by a research nurse. As data-enabled tablets and training were provided to trial participants, there were no specific technical prerequisites for inclusion regarding experience with or ownership of smartphones or tablets.

Dyads in the control group received standard care only, representing the routine health care services provided outside the trial. Care varied across participants depending on several factors, including general health, comorbidities, and health-seeking behavior. In Sweden, standard care for older adults generally includes an annual in-person physician visit for routine check-ups or a telephone consultation to discuss overall health and, if necessary, annual laboratory tests and prescription renewals. In Spain, standard care generally includes appointments at a health center, where diagnostic, therapeutic, and preventive services are provided, along with referrals to specialist services when needed [27].

### Outcome Measures

The primary outcome of the trial was QoL, measured using the QoL-AD score for PwMCI at the 18-month follow-up. Other outcomes were treated as secondary outcome measures and categorized into confirmatory and exploratory end points to address multiplicity concerns and maintain statistical rigor. Confirmatory outcome measures for PwMCI included health-related QoL measured using the EQ-5D-3L, global cognitive function measured using MMSE scores [25], and medication adherence. Caregiver burden, measured using the Zarit Burden Interview (ZBI) score, was included as a confirmatory outcome for informal caregivers. All remaining outcome measures, subgroup analyses, and sensitivity analyses were considered exploratory.

Initially developed for persons with Alzheimer disease, the QoL-AD is a disease-specific instrument used to measure QoL, although its application has since expanded to other dementia disorders [28]. The scale consists of 13 items assessing physical health, energy, mood, living situation, memory, family, marriage, friends, self as a whole, ability to do chores, ability to engage in enjoyable activities, financial situation, and life as a whole. Responses are rated as 1 (poor), 2 (fair), 3 (good), or 4 (excellent), and summed to generate a total score, with higher scores indicating better QoL [29]. Ratings from PwMCI were obtained through interviews using standardized instructions, whereas caregiver ratings were obtained through a questionnaire. A weighted composite score was calculated by combining the PwMCI and informal caregiver ratings of the PwMCI’s QoL, with twice the weight assigned to the PwMCI’s self-assessment, following Logsdon et al [30].

The EQ-5D-3L questionnaire is a generic preference-based instrument used to measure quality-adjusted life years (QALYs). It consists of 5 domains: mobility, self-care, usual activities, pain/discomfort, and anxiety/depression. Each domain has 3 levels: no problems, some problems, and extreme problems, resulting in 243 possible health states [31]. These health states are converted into a score ranging from 0 (equivalent to death) to 1 (perfect health) using population preference values, commonly referred to as “tariffs” [32]. As tariffs differ across countries, the use of country-specific tariffs is recommended when calculating QALYs [33].

This study used the European EQ-5D Visual Analogue Scale (EVAS) tariff, which aggregates health state valuations from 6 EuroQol Group countries, including Spain and Sweden. This single European value set provides a standardized approach to measuring health utility across diverse populations. A key justification for this approach is the substantial agreement in EQ-5D VAS health state valuations across diverse European populations, supporting the use of a unified value set for cross-country comparisons [34]. Although the EVAS measures health-related QoL on a scale ranging from 0 (worst imaginable health) to 100 (best imaginable health), health utility scores for QALY calculation must be anchored between 0 (dead) and 1 (full health). To meet this requirement, we rescaled the EVAS scores into health utility weights for QALY calculation following the method described by Greiner et al [34]. The following formula was applied to convert the 100-point scale to a 0-1 utility index.

VAS_res_=(VAS*_i_*–VAS_dead_median_)/(VAS_11111_–VAS_dead_median_)

where VAS*_i_* is the raw score (0-100) given by the respondent for a specific health state; VAS_dead_median_ is the raw score the respondent gave to the state “dead”; and VAS_11111_ is the raw score the respondent gave to “best imaginable health.”

Furthermore, we used the median value for death (2.0) rather than the mean value (5.3) for rescaling, based on the statistics reported in Table 4 of Greiner et al [34]. This choice was made to ensure that the anchors reflected the valuation of the typical respondent, as the median is more robust to skewed distributions and extreme outliers commonly observed in VAS data. In addition, using the median more accurately represents the consensus within the European sample that the worst health state is perceived as only marginally better than death. For example, if a patient’s VAS score for a specific health state was 78, then:

VAS*_i_*=78; VAS_dead_median_=2; VAS_11111_=97.66.

The resulting rescaled EVAS value is 0.7944 based on the aforementioned formula.

These scores were used to calculate accumulated QALYs over the trial period using the area-under-the-curve approach [35].

The MMSE is a standardized test of cognitive function in older adults that assesses memory, attention, orientation, visuospatial skills, and language [25]. The maximum MMSE score is 30, whereas a score lower than 24 indicates dementia; however, the cutoff score for MCI has not been consistently established [36]. The Instrumental Activities of Daily Living (IADL) is a validated tool used to assess the functional abilities of older adults in performing tasks necessary for independent living [37]. These tasks include shopping, housekeeping, meal preparation, laundry, independent travel, financial management, and medication management [37]. The summary score ranges from 0 (low function, dependent) to 8 (high function, independent) [37].

The outcome measures for informal caregivers were QALYs and caregiver burden assessed using the short-form 12-item ZBI (ZBI-12). The ZBI-12 is a 12-item self-report instrument that measures the impact of informal caregiving on the caregiver’s health and social life [38]. Each item is scored on a 5-point Likert scale ranging from “never” to “almost always,” with a total score ranging from 0 to 48, where scores of 17 or higher indicate a high caregiving burden [38].

### Medication Adherence

At baseline, participating PwMCI registered up to 10 long-term prescribed medications, defined as medications intended to be taken for at least 6 months. Medication adherence was assessed for the 30 days preceding each follow-up visit for the 2 most frequently taken medications in tablet or capsule form (U Isaksson and J Frögen, unpublished data, 2018). A research nurse contacted participating PwMCI by telephone at 5, 11, and 17 months to confirm that they were taking the same 2 medications identified at baseline or to update the database if changes had occurred in their long-term prescribed medications. Dyads were asked to bring empty medication packaging used during the previous 30 days, remaining pills (or pill equivalents), and documents related to prescription history to the follow-up visits. Follow-up assessments at 6, 12, and 18 months were conducted for participants in both groups by research nurses using pill counts (number of pills taken). PwMCI who experienced changes in their medication regimen (eg, dose or frequency) were excluded from adherence calculations to prevent regimen modifications from biasing the results.

Overall medication adherence was measured by dividing the number of pills taken by the number of pills prescribed for each of the 2 drugs separately and expressing the result as a percentage. This percentage variable was then transformed into a binary variable representing overall adherence, where adherence rates between 80% and 110% were considered adherent, as described by Anderberg et al [20]. The composite measure of overall adherence for both drugs classified PwMCI as adherent only if they were adherent to both medications. PwMCI who were adherent to only 1 drug or who were prescribed only 1 drug were classified as nonadherent. Therefore, PwMCI prescribed only 1 drug were included only in the supplementary analysis of “change in medication adherence for at least one drug” and excluded from the analysis of “change in medication adherence for both drugs” to avoid measurement bias.

### Statistical Analyses

The primary analysis followed a modified intention-to-treat approach, including randomized dyads with available outcome data at the relevant follow-up. Missing outcome data were not imputed in the primary analysis and were addressed in sensitivity analyses. Baseline characteristics were compared between the intervention and control groups to confirm balance achieved through randomization. We also performed a dropout analysis at 18 months by comparing baseline characteristics between dropouts and nondropouts in the intervention and control groups.

Repeated measurements were collected at multiple time points (baseline, 6, 12, and 18 months), so differences between the intervention and control groups for each outcome were analyzed using mixed-effects models [39]. Results are presented as means with SEs and mean differences with 95% CIs. We fitted mixed-effects models including intervention group (SMART4MD vs control) and time (baseline, 6, 12, and 18 months) as fixed effects, with a random intercept for participants (clustered within group). The overall intervention effect and the interaction between intervention and time were used to estimate the differential impact of SMART4MD app use on outcomes over time. Mixed-effects logistic regression models were used to estimate medication adherence among PwMCI. In a supplementary analysis, we also fitted mixed-effects models to estimate adherence to at least one drug. All models were adjusted for study sites (BTH, CST, and SAS).

Statistical significance was set at a 2-sided *P* value <.05. Data analysis was performed using Stata/SE 15.1 software (StataCorp LP).

In this study, QALY was calculated for each follow-up assessment (6, 12, and 18 months). In the SMART4MD trial, an 180-day window with a tolerance of +18 or –18 days was allotted for conducting each visit. However, as is often the case in trials, some participant visits occurred outside the required 180-day window period. Hence, instead of relying on the predetermined 180-day interval, we used the actual number of days between 2 visits to calculate QALY at each follow-up assessment. To determine the total QALYs for the entire trial period (18 months), we summed the QALYs obtained at each follow-up assessment and excluded participants who missed any follow-up assessment.

For dyad-level QALYs, PwMCI and caregiver QALYs were summed to represent the total dyadic health benefit, and results are presented as mean (SD) or mean differences.

Results for exploratory analyses are reported with 95% CIs and nominal *P* values without formal adjustment for multiplicity and should therefore be interpreted as hypothesis-generating. Multiplicity adjustment for confirmatory outcomes was applied separately for PwMCI (medication adherence, MMSE, and EQ-5D-3L) and informal caregivers (ZBI scores) using the Holm-Bonferroni method [40].

### Sensitivity and Subgroup Analyses

#### Assessment of Effect Modification

Several sensitivity and subgroup analyses were performed, considering that the effect of the intervention might differ according to participant characteristics [41].

#### Sensitivity Analyses

##### Overview

We conducted sensitivity analyses for QoL-AD scores based on imputed QoL-AD scores, δ-adjusted tipping-point sensitivity analyses for QoL-AD and QALY, PwMCI-assessed QoL-AD scores, caregiver-assessed QoL-AD scores, user behavior, and the method described in the protocol paper (Monte Carlo permutation *t* test) [20].

##### Multiple Imputations for Missing QoL-AD

We used multiple imputation to impute missing information on the composite QoL-AD score for both groups [42], generating 20 different datasets. The baseline variables used in the models were age, sex, education, living arrangements, composite QoL-AD score, EQ-5D-3L index score, MMSE score, ZBI score, and intervention/control group. We then analyzed the multiple datasets and computed pooled estimates following the methods described by Rubin [43].

##### δ-Adjusted Tipping-Point Sensitivity Analyses for QoL-AD and QALY

To assess the robustness of the findings for the primary outcome (QoL-AD) to potential missing-not-at-random mechanisms, we conducted δ-adjusted tipping-point sensitivity analyses at the 18-month follow-up. Further, δ-adjusted tipping-point sensitivity analyses were also performed for the imputed QALY of PwMCI and informal caregivers at the 18-month follow-up. We systematically applied a range of departures from the missing-at-random assumption by adding a constant (δ) to the imputed outcome values in both groups. The tipping point was defined as the value of δ at which the treatment effect either lost statistical significance (*P*≥.05) or required a shift in outcomes judged to be clinically implausible.

##### User Behavior

Participants were grouped based on high (>125 launches) or low (≤125 launches) app use to observe the impact of app usage on QoL. We measured SMART4MD app use by counting the number of times the app was launched during the first 12-month period (data on app use for the last 6 months were unavailable). We created a binary variable using the median cutoff of 125 launches.

##### QoL-AD Analysis According to the Protocol

In the protocol paper [20], the primary outcome was defined as the mean change in total QoL-AD score at 18 months. The planned analysis involved a 2-group comparison of this mean change using a Monte Carlo permutation *t* test, with bootstrap methods applied to estimate 95% CIs for the difference between group means.

#### Subgroup Analyses

##### Overview

Interaction term analyses were conducted to evaluate differences in the effect of the intervention across subgroups based on demographic characteristics (sex and age) and cognitive function (MMSE scores) on QoL-AD scores.

##### Sex Comparison

Changes in QoL-AD scores were compared between men and women.

##### Age Comparison

Participants were categorized into 2 groups, those aged up to 70 years and those aged over 70 years, to assess differences in QoL-AD scores [44,45].

##### Cognitive Status

Participants were divided based on baseline MMSE score (≤26 vs >26) to evaluate the effect of cognitive function on QoL, as previous studies suggested that this cutoff is preferable because of its better specificity and sensitivity [41,46]. Additionally, this cutoff represented the median in our sample.

We also explored sensitivity analyses for health outcomes and medication adherence based on study sites. Further, the sensitivity and interaction analyses based on QALY (EVAS) also incorporated multiple imputation for QALY (EVAS) scores across all time points (baseline, 6, 12, and 18 months). For medication adherence, we conducted a sensitivity analysis defining adherence as adherence to at least one drug. Within-group (intragroup) differences in health outcomes were also assessed using paired *t* tests. The percentages of missing and imputed values were calculated for both QoL-AD scores and QALY at each data collection point. Results for all these analyses are presented in Multimedia Appendix 2.

## Results

### Participant Baseline Characteristics

**Figure 1:**
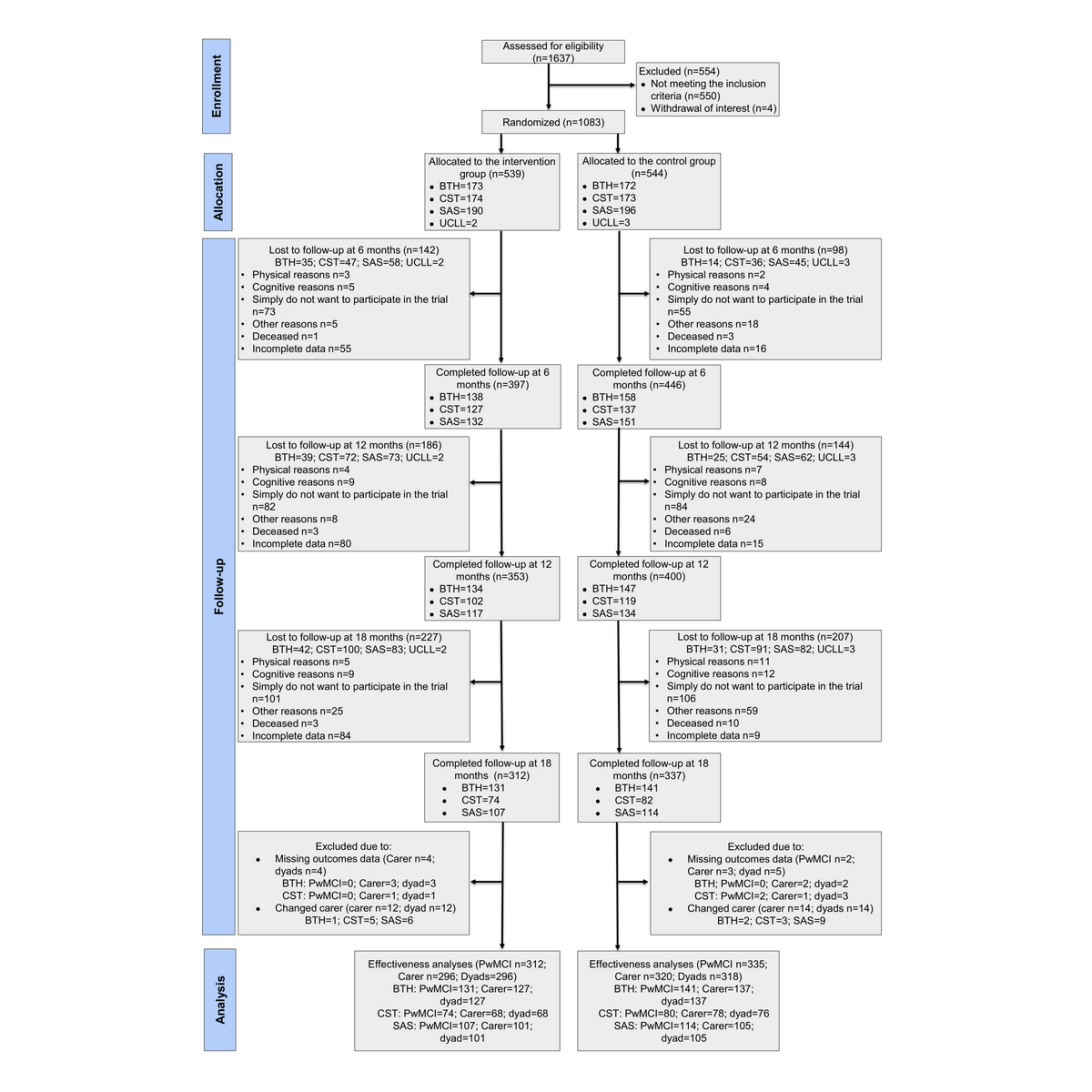
CONSORT (Consolidated Standards of Reporting Trials) flowchart. BTH: Blekinge Institute of Technology; CST: Consorci Sanitaria de Terrassa; SAS: Servicio Andaluz de Salud; UCLL: University College Leuven-Limburg.

The baseline demographic characteristics of PwMCI and informal caregivers in the intervention and control groups are presented in Table 1. Figure 1 presents the study flowchart.

**Table 1:** Demographic characteristics of PwMCI^a^ and their informal caregivers at baseline.

Characteristics		PwMCI^b^					Informal caregiver^c^				
		Intervention (n=537)	Control (n=541)	*P* value		Intervention (n=537)		Control (n=541)		*P* value	
Age (years), mean (SD)		75 (7.24)	74 (7.23)	.47		62 (15.00)		63 (14.36)		.33	
**Sex, n (%)**					.18						.48
	Male	241 (44.9)	265 (49.0)			166 (30.9)		178 (32.9)			
	Female	296 (55.1)	276 (51.0)			371 (69.1)		363 (67.1)			
**Education, n (%)**					.17						.09
	Elementary education	313 (58.3)	333 (61.6)			175 (32.6)		201 (37.2)			
	Secondary education	124 (23.1)	100 (18.5)			186 (34.6)		156 (28.8)			
	Higher education	97 (18.1)	106 (19.6)			161 (30.0)		173 (32.0)			
**Civil status, n (%)**					.13						.39
	Single	181 (33.7)	159 (29.4)			105 (19.6)		95 (17.6)			
	Married/living together	356 (66.3)	381 (70.4)			430 (80.1)		445 (82.3)			
**Living arrangements, n (%)**					.16						.65
	Single	117 (21.8)	102 (18.9)			52 (9.7)		43 (7.9)			
	Spouse/common law partner	327 (60.9)	356 (65.8)			357 (66.5)		378 (69.9)			
	Children	56 (10.4)	41 (7.6)			53 (9.9)		48 (8.9)			
	Other	32 (6.0)	39 (7.2)			72 (13.4)		72 (13.3)			

^a^PwMCI: people with mild cognitive impairment.

^b^Missing data for education (intervention, n=3; control, n=2); civil status (intervention, n=0; control, n=1); living arrangements (intervention, n=5; control, n=3)

^c^Missing data for education (intervention, n=15; control, n=11); civil status (intervention, n=2; control, n=1); living arrangements (intervention, n=3; control, n=0).

### Effect of the Intervention on Health Outcomes

#### PwMCI

##### Composite Quality of Life (QoL-AD)

The mixed-effects model revealed a positive impact of the intervention on the primary outcome of QoL (QoL-AD). At 6 months, there was a significant mean difference between the intervention and control groups (mean difference 0.73, 95% CI 0.09-1.36; *P*=.02). This significant difference in QoL-AD score was sustained at the 18-month follow-up (mean difference 0.75, 95% CI 0.07-1.42; *P*=.03; Table 2).

**Table 2:** Differences in outcome measures between intervention and control groups for PwMCI^a^.

		Sample size (intervention/control), n/N	Intervention group, mean (SE)	Control group, mean (SE)	Difference^b^, mean (95% CI)	*P* value
**Composite QoL-AD^c^**						
	Baseline	516/517	36.01 (0.22)	35.89 (0.22)	0.122 (–0.47 to 0.72)	.69
	Month 6	375/426	36.62 (0.23)	35.89 (0.23)	0.729 (0.09 to 1.36)	.02
	Month 12	259/294	36.25 (0.25)	35.73 (0.24)	0.521 (–0.17 to 1.21)	.14
	Month 18	289/314	36.30 (0.25)	35.55 (0.24)	0.746 (0.07 to 1.42)	.03
**EQ-5D-3L index scores (EVAS^d^)**						
	Baseline	536/538	0.772 (0.009)	0.774 (0.009)	–0.002 (–0.03 to 0.02)	.85
	Month 6	395/441	0.763 (0.010)	0.752 (0.009)	0.011 (–0.01 to 0.04)	.41
	Month 12	350/396	0.758 (0.010)	0.758 (0.009)	0.0005 (-0.03 to 0.03)	.97
	Month 18	311/334	0.758 (0.010)	0.752 (0.010)	0.005 (–0.02 to 0.03)	.71
**Total accumulated QALY^e^ (EVAS)^f^**						
	Month 6	395/442	0.423 (0.006)	0.388 (0.005)	0.035 (0.02 to 0.05)	<.001
	Month 12	340/383	0.365 (0.006)	0.380 (0.006)	–0.015 (–0.03 to 0.00)	.08
	Month 18	298/334	0.365 (0.006)	0.353 (0.006)	0.012 (–0.01 to 0.03)	.17
**MMSE^g^**						
	Baseline	537/538	25.51 (0.14)	25.63 (0.14)	–0.117 (–0.49 to 0.26)	.54
	Month 6	394/440	25.50 (0.15)	25.51 (0.14)	–0.009 (–0.42 to 0.40)	.97
	Month 12	352/398	25.19 (0.15)	25.50 (0.15)	–0.309 (–0.73 to 0.11)	.15
	Month 18	310/335	25.43 (0.16)	25.26 (0.16)	0.169 (–0.27 to 0.61)	.45
**IADL^h,f^**						
	Baseline	471/484	6.71 (0.08)	6.65 (0.08)	0.060 (–0.17 to 0.29)	.60
	Month 6	363/405	6.17 (0.09)	6.19 (0.09)	–0.022 (–0.26 to 0.22)	.86
	Month 12	330/330	6.00 (0.09)	6.11 (0.09)	–0.110 (–0.36 to 0.14)	.39
	Month 18	292/312	5.83 (0.09)	5.88 (0.09)	–0.049 (–0.30 to 0.21)	.70

^a^PwMCI: people with mild cognitive impairment.

^b^Between-group differences are computed using a mixed-effect model, and results are controlled for site.

^c^QoL-AD: Quality of Life in Alzheimer’s Disease.

^d^EVAS: European EQ-5D Visual Analogue Scale.

^e^QALY: quality-adjusted life years.

^f^*P* values for exploratory outcomes are nominal and have not been adjusted for multiple comparisons.

^g^MMSE: Mini-Mental State Examination.

^h^IADL: Instrumental Activities of Daily Living.

##### EQ-5D-3L Index Scores (EVAS), MMSE, and IADL Scores

By contrast, no significant differences were found between the 2 groups in health utility (EQ-5D-3L, 6 months: *P*=.41; 12 months: *P*=.97; and 18 months: *P*=.71), cognitive function (MMSE, 6 months: *P*=.97; 12 months: *P*=.15; and 18 months: *P*=.45), or activities of daily living (IADL, 6 months: nominal *P*=.86; 12 months: nominal *P*=.39; and 18 months: nominal *P*=.70) at any follow-up interval (Table 2).

##### Total Accumulated QALY

Although accumulated QALY was significantly different at 6 months (mean difference 0.035, 95% CI 0.02-0.05; nominal *P*<.001), this effect was not sustained at the 12- and 18-month follow-ups (Table 2).

#### Informal Caregivers and Dyads

##### EQ-5D-3L Index Scores for Informal Caregivers

For informal caregivers, the intervention was associated with a significant improvement in health utility (EQ-5D-3L) at the 18-month follow-up (mean difference 0.030, 95% CI 0.001-0.058; nominal *P*=.04), although there were no significant differences at earlier time points (6 months: nominal *P*=.15 and 12 months: nominal *P*=.69). Despite this improvement, there was no significant impact on caregiver burden, as measured by the ZBI, at any point during the study (6 months: *P*=.96; 12 months: *P*=.60; and 18 months: *P*=.32; Table 3).

**Table 3:** Differences in outcome measures between intervention and control groups for informal caregivers and dyad.

Differences				Sample size (intervention/control), n/N		Intervention group, mean (SE)		Control group, mean (SE)		Difference^a^		
										Mean (95% CI)		*P* value
**Informal caregivers**												
	**EQ-5D-3L index scores (EVAS^b^)^c^**											
		Baseline	519/518		0.80 (0.009)		0.79 (0.009)		0.013 (–0.01 to 0.04)		.26	
		Month 6	376/427		0.81 (0.010)		0.79 (0.009)		0.019 (–0.007 to 0.045)		.15	
		Month 12	331/371		0.79 (0.010)		0.78 (0.010)		0.006 (–0.02 to 0.03)		.69	
		Month 18	296/320		0.80 (0.010)		0.77 (0.010)		0.030 (0.001 to 0.058)		.04	
	**Total accumulated QALY^d^ (EVAS)^c^**											
		Month 6	375/422		0.448 (0.006)		0.398 (0.005)		0.050 (0.04 to 0.07)		<.001	
		Month 12	314/354		0.388 (0.006)		0.397 (0.006)		–0.009 (–0.03 to 0.01)		.26	
		Month 18	279/303		0.385 (0.006)		0.376 (0.006)		0.009 (–0.01 to 0.03)		.31	
	**ZBI^e^ scores**											
		Baseline	520/521		6.59 (0.32)		6.78 (0.32)		–0.183 (–1.08 to 0.71)		.69	
		Month 6	378/427		6.74 (0.36)		6.76 (0.34)		–0.023 (–0.99 to 0.95)		.96	
		Month 12	332/370		6.59 (0.37)		6.86 (0.36)		–0.270 (–1.28 to 0.74)		.60	
		Month 18	292/318		7.06 (0.38)		7.59 (0.37)		–0.531 (–1.58 to 0.52)		.32	
**Dyads (PwMCI^f^ plus informal caregivers)**												
	**Total accumulated QALY (EVAS)^c^**											
		Month 6	372/420		0.874 (0.009)		0.789 (0.009)		0.085 (0.06 to 0.11)		<.001	
		Month 12	312/350		0.759 (0.010)		0.781 (0.009)		–0.022 (–0.05 to 0.005)		.11	
		Month 18	276/300		0.758 (0.011)		0.739 (0.010)		0.018 (–0.01 to 0.05)		.21	

^a^Between-group differences are computed using the mixed-effect model and results are controlled for site.

^b^EVAS: European EQ-5D Visual Analogue Scale.

^c^*P* values for exploratory outcomes are nominal and have not been adjusted for multiple comparisons.

^d^QALY: quality-adjusted life years.

^e^ZBI: Zarit Burden Interview.

^f^PwMCI: people with mild cognitive impairment.

##### Total Accumulated QALY for Informal Caregivers

At the 6-month follow-up, there were significant differences in total accumulated QALY (mean difference 0.050, 95% CI 0.04-0.07; nominal *P*<.001), but this effect did not persist at later follow-ups (Table 3).

While evaluating the dyads, the intervention group showed a significant increase in total accumulated QALY at the 6-month follow-up (mean difference 0.085, 95% CI 0.06-0.11; nominal *P*<.001). This initial benefit did not reach statistical significance at the 12- (nominal *P*=.11) or 18-month (nominal *P*=.21) follow-up (Table 3).

### Effect of Intervention on Medication Adherence

There were no significant differences in medication adherence between groups at the 6-, 12-, or 18-month follow-ups (Table 4). At the 18-month follow-up, the estimated probability of adhering to both prescribed drugs was slightly higher in the SMART4MD group than in the control group; however, this difference was not statistically significant (absolute difference in probabilities=0.09; *P*=.12). The results for medication adherence to at least one drug also showed no differences between groups (see Table S1 in Multimedia Appendix 2).

**Table 4:** Change in medication adherence (≥80% and ≤110%) between the intervention and control group for both drugs at 6-, 12-, and 18-month follow-up using the logit model.

Period	Sample size (intervention/control), n/N	Intervention group, mean (SE)	Control group, mean (SE)	Absolute difference^a^ in probabilities	
				Difference (95% CI)	*P* value
Month 6	187/221	0.422 (0.04)	0.395 (0.03)	0.027 (–0.07 to 0.12)	.58
Month 12	123/160	0.447 (0.04)	0.485 (0.04)	–0.038 (–0.15 to 0.08)	.53
Month 18	139/157	0.474 (0.04)	0.385 (0.04)	0.090 (–0.02 to 0.20)	.12

^a^Between-group differences are computed using multilevel mixed-effect logistic regression (melogit) and results are controlled for site.

Overall, the intervention showed benefits for PwMCI in terms of composite QoL-AD scores and nominally significant benefits for informal caregivers in terms of EQ-5D-3L index scores at 18 months (nominal *P*=.04)); however, no significant differences were found for the dyads at 18 months (nominal *P*=.21). There was no significant impact on medication adherence (6 months: *P*=.58; 12 months: *P*=.53; and 18 months: *P*=.12). All confirmatory outcomes for PwMCI (medication adherence, MMSE score, and EQ-5D-3L) and informal caregivers (ZBI scores) were nonsignificant before adjustment and remained so after Holm-Bonferroni correction (all *P*_adj_>.99).

### Sensitivity and Subgroup Analyses

#### Greater QoL-AD Scores in the Intervention Group

Table 5 shows the sensitivity analyses for the primary outcome (QoL-AD). The findings remained robust when using imputed QoL-AD scores to account for missing values. A significant improvement was observed at 6 months, with a mean difference of 0.708 (nominal *P*=.01). Consistent with the base-case results, no significant difference was found in imputed QoL-AD scores at the 12-month follow-up (nominal *P*=.16). However, results were significantly higher for the intervention group at 18 months (nominal *P*=.02). Similarly, the results for PwMCI-assessed QoL-AD scores were significantly higher at 6 months (nominal *P*=.04). However, at the 12-month (nominal *P*=.38) and 18-month (nominal *P*=.14) follow-ups, there were no significant differences between the 2 groups. The sensitivity analysis for caregiver-assessed QoL-AD scores found no significant differences between the intervention and control groups (6 months: nominal *P*=.08; 12 months: nominal *P*=.20; and 18 months: nominal *P*=.08). High app usage (>125 launches) had a significant effect on composite QoL-AD scores at the 6-month (nominal *P*=.01) and 18-month (nominal *P*=.04) follow-ups, but not at the 12-month follow-up (nominal *P*=.07). However, the model did not find significant effect for low app usage (≤125 launches) at any time point (6 months: nominal *P*=.18; 12 months: nominal *P*=.56; and 18 months: nominal *P*=.15). The sensitivity analysis using a Monte Carlo permutation *t* test showed no statistically significant difference between the 2 groups in change in QoL-AD scores at the 18-month follow-up (*P*=.45).

**Table 5:** Sensitivity analyses for the primary outcome QoL-AD^a^ for PwMCI^b^.

Case		Sample size (intervention/control), n/N	Intervention group, mean (SE)	Control group, mean (SE)	Difference^c^, mean (95% CI)	*P* value
**Base case**						
	Baseline	516/517	36.01 (0.22)	35.89 (0.22)	0.122 (–0.47 to 0.72)	.69
	Month 6	375/426	36.62 (0.23)	35.89 (0.23)	0.73 (0.09 to 1.36)	.02
	Month 12	259/294	36.25 (0.25)	35.73 (0.24)	0.52 (–0.17 to 1.21)	.14
	Month 18	289/314	36.30 (0.25)	35.55 (0.24)	0.75 (0.71 to 1.42)	.03
**Imputed composite QoL-AD score^d^**						
	Baseline	520/520	35.81 (0.21)	35.70 (0.21)	0.108 (–0.46 to 0.68)	.71
	Month 6	519/521	36.44 (0.21)	35.73 (0.21)	0.708 (0.14 to 1.28)	.01
	Month 12	390/439	36.11 (0.22)	35.68 (0.21)	0.427 (–0.17 to 1.02)	.16
	Month 18	348/389	36.11 (0.22)	35.38 (0.22)	0.731 (0.12 to 1.34)	.02
**PwMCI-assessed QoL-AD score^d^**						
	Baseline	536/538	36.57 (0.23)	36.40 (0.23)	0.164 (–0.47 to 0.80)	.61
	Month 6	395/442	37.14 (0.25)	36.43 (0.24)	0.702 (0.02 to 1.38)	.04
	Month 12	296/341	36.70 (0.27)	36.38 (0.26)	0.325 (–0.40 to 1.05)	.38
	Month 18	312/334	36.73 (0.26)	36.19 (0.26)	0.547 (–0.18 to 1.27)	.14
**Carer-assessed QoL-AD score^d^**						
	Baseline	517/519	34.73 (0.26)	34.85 (0.26)	–0.119 (–0.84 to 0.60)	.74
	Month 6	377/428	35.46 (0.28)	34.78 (0.27)	0.684 (–0.09 to 1.46)	.08
	Month 12	269/302	35.25 (0.31)	34.70 (0.30)	0.550 (–0.29 to 1.39)	.20
	Month 18	289/317	35.19 (0.30)	34.46 (0.29)	0.733 (–0.10 to 1.56)	.08
**High app use >125^d^**						
	Baseline	168/517	35.98 (0.37)	35.95 (0.22)	0.030 (–0.82 to 0.88)	.94
	Month 6	165/426	37.10 (0.38)	35.96 (0.22)	1.140 (0.28 to 2.00)	.009
	Month 12	135/294	36.63 (0.39)	35.79 (0.24)	0.835 (–0.07 to 1.74)	.07
	Month 18	167/314	36.54 (0.38)	35.62 (0.24)	0.924 (0.05 to 1.80)	.04
**Low app use ≤125^d^**						
	Baseline	169/517	36.20 (0.38)	35.92 (0.22)	0.281 (–0.58 to 1.14)	.52
	Month 6	167/426	36.51 (0.38)	35.92 (0.23)	0.592 (–0.28 to 1.46)	.18
	Month 12	116/294	36.04 (0.41)	35.76 (0.24)	0.277 (–0.66 to 1.21)	.56
	Month 18	118/314	36.26 (0.41)	35.58 (0.24)	0.681 (–0.25 to 1.61)	.15
**Monte Carlo permutation *t* test, mean (SD)**						
	QoL‑AD total score at 18 months^e^	310/328	36.51 (5.16)	36.02 (5.03)	0.32 (–0.50 to 1.14)^f^	.45^g^

^a^QoL-AD: Quality of Life in Alzheimer’s Disease.

^b^PwMCI: people with mild cognitive impairment.

^c^Between-group differences are computed using the mixed-effect model and results are controlled for site.

^d^*P* values for exploratory outcomes are nominal and have not been adjusted for multiple comparisons.

^e^The number of permutations is 10,000, and the number of bootstrap replications is 10,000.

^f^95% bootstrap CI for the difference in mean QoL-AD score between the intervention and control groups (intervention minus control).

^g^Two-sample, 2-sided Monte Carlo permutation *t* test.

#### Interaction Analyses

##### Overview

Table S2 in Multimedia Appendix 2 shows the interaction terms for sex, age, and MMSE scores in relation to QoL-AD scores.

##### Sex

The effect of the SMART4MD app appeared to be slightly greater for women than for men; however, the difference was not statistically significant (nominal *P*=.82).

##### Age

A nonsignificant negative interaction was observed for PwMCI aged over 70 years compared with those aged 70 years or under (nominal *P*=.67).

##### Cognitive Status (MMSE Score)

Similarly, no significant interaction was observed between cognitive function (MMSE >26 vs ≤26) and QoL-AD scores (nominal *P*=.12).

##### Demographic Characteristics at the 18-Month Follow-Up

No significant differences were observed between the intervention and control groups (all *Ps*>.05, exact *P* values are presented in Table S3 in Multimedia Appendix 2), except for living arrangements at the 18-month follow-up, where both PwMCI (73/312, 23.4%; *P*=.01) and informal caregivers (27/296, 9.1%; *P*=.04) in the intervention group were more likely to live alone (Table S3 in Multimedia Appendix 2).

#### Within-Group Differences in Effect Measures

There were no significant differences in QoL-AD scores (intervention: *P*=.18; control: *P*=.09) from baseline to 18-month follow-up in either group (Table S4 in Multimedia Appendix 2), although both groups experienced a significant reduction in IADL scores (nominal *P*<.001) among PwMCI. Over the trial period, a statistically significant reduction in EQ-5D-3L index scores was observed in the control group (mean difference –0.028; *P*=.006), but not in the intervention group (*P*=.08) for PwMCI. No significant differences in EQ-5D-3L index scores (intervention: nominal *P*=.43; control: nominal *P*=.24) or ZBI scores (intervention: *P*=.31; control: *P*=.14) from baseline to follow-up were observed in either group of informal caregivers. EQ-5D-3L index scores for dyads were also significantly reduced in the control group over the trial period (mean difference –0.048; nominal *P*=.006), but not among dyads in the intervention group (nominal *P*=.11; see Table S4 in Multimedia Appendix 2).

#### Sensitivity and Interaction Analyses Using QALY (EVAS)

Sensitivity analyses using QALYs based on the EVAS tariff as the outcome are presented in Table S5 in Multimedia Appendix 2. Significant differences were observed in imputed QALYs for PwMCI, showing a significant improvement in the intervention group compared with the control group at 6-month follow-up (nominal *P*<.001). Interaction analyses for QALYs (EVAS) did not indicate any heterogeneity in the intervention effect across groups based on age, sex, and cognitive status (Table S6 in Multimedia Appendix 2).

For informal caregivers, the general base-case results were consistent, with statistically significant results for imputed QALY (*P*=.02) showing better QoL in the intervention group at the 18-month follow-up (Table S7 in Multimedia Appendix 2). Further, interaction analyses for QALY (EVAS) among informal caregivers did not show significant differences based on age (*P*=.40), sex (*P*=.37), and caregiver burden (*P*=.33; see Table S6 in Multimedia Appendix 2).

The sensitivity analysis based on imputed QALY showed a significant improvement in QoL for dyads in the intervention group compared with those in the control group at 6 months (*P*<.001) and 18 months (*P*=.01; see Table S7 in Multimedia Appendix 2).

#### Health Outcomes and Medication Adherence Based on Study Sites

Results for intergroup differences in health outcomes and medication adherence across the 3 sites (BTH, CST, and SAS) are presented in Tables S8-S10 in Multimedia Appendix 2, respectively. These findings showed significantly higher MMSE scores (*P*=.02) at the 18-month follow-up and higher QALY scores (*P*<.001) for PwMCI at the 6-month follow-up for the SAS site (Table S8 in Multimedia Appendix 2). For dyads, QALY scores were also significantly higher in the intervention group than in the control group at 6-month follow-up (*P*=.003) and 18-month follow-up (*P*=.03) at the CST site and at 6-month follow-up (*P*<.001) at the SAS site (Table S9 in Multimedia Appendix 2). At the SAS site, at the 6-month follow-up, the probability of adhering to both prescribed drugs was significantly higher in the SMART4MD group than in the control group (absolute difference in probabilities .13; nominal *P*=.02; Table S10 in Multimedia Appendix 2).

### Attrition

Attrition refers to the exclusion of participants from the final analysis, usually because of factors such as dropout from the RCT or missing data. The failure rate of dyads to respond to all health outcomes at the 18-month follow-up was 41.9% (225/537) in the intervention group and 37.7% (204/541) in the control group (*P*=.16). The stated reasons for exiting the trial were predominantly related to a general unwillingness to continue participation in the trial. PwMCI who dropped out from the intervention group were significantly more likely to be older (*P*<.001), female (*P*=.005), unmarried (*P*=.024), and less likely to live with a spouse (*P*=.02). They also tended to have worse MMSE and IADL scores at baseline. In the control group, PwMCI who dropped out were less likely to live with a spouse, less likely to have a high level of education, and had worse QoL-AD, MMSE, and IADL scores at baseline (Table S11 in Multimedia Appendix 2). The characteristics of informal caregivers among the dropouts are presented in Table S12 in Multimedia Appendix 2. These caregivers tended to be younger, less likely to live with a spouse, and to have a higher caregiver burden at baseline. At 18 months, multiple imputation under missing at random for imputed QoL-AD scores (mean difference 0.731; nominal *P*=.02), imputed QALY for PwMCI (mean difference 0.010; nominal *P*=.13), and imputed QALY for informal caregivers (mean difference 0.014; nominal *P*=.02) yielded similar estimates in tipping-point analyses applying δ shifts (Table S13 in Multimedia Appendix 2).

For PwMCI, the highest percentage of missing values (501/1078, 46.5%) was observed for QoL-AD scores at the 12-month follow-up (Table S14 in Multimedia Appendix 2). By contrast, for QALY, the highest percentages of missing values were nearly 40% for PwMCI (433/1078, 40.2%) and informal caregivers (436/1078, 40.4%) at the 18-month follow-up (Table S14 in Multimedia Appendix 2).

## Discussion

### Principal Findings

This study investigated whether the SMART4MD intervention improved QoL and medication adherence among PwMCI, as well as reduced caregiver burden during the 18-month trial period. Significant differences in QoL-AD scores were observed in the intervention group, and this finding remained robust in the sensitivity analysis using imputed composite QoL-AD scores. The model demonstrated a significant interaction between the intervention and time. The results fluctuated because no significant differences were observed at the 12-month follow-up; however, consistent differences in scores were found at the 6- and 18-month follow-ups. The SMART4MD intervention did not demonstrate a significant impact on the confirmatory outcomes for PwMCI (medication adherence, MMSE, and EQ-5D-3L) or informal caregivers (ZBI scores), with results remaining nonsignificant after controlling for multiplicity. Although exploratory results for EQ-5D-3L index scores were significantly different for informal caregivers in the intervention group, no significant improvement was found in total accumulated QALY at the 18-month follow-up. Sensitivity analyses based on imputed QoL-AD scores indicated significantly better QoL for PwMCI in the intervention group. The SMART4MD app includes reminders and other features intended to improve medication adherence; however, the study found that these features did not lead to a significant improvement in medication adherence. It is important to consider potential factors that might influence the outcomes, such as sex, age, cognitive status, and caregiver burden. However, the study did not find significant interaction effects on QoL-AD based on these factors that could explain the lack of improvement in QoL-AD and medication adherence.

QoL-AD scores were higher in the intervention group when imputed QoL-AD scores were used to account for missing data through multiple imputation. This suggests that the positive effect of the intervention on QoL-AD is robust and not substantially affected by missing data. In addition, QoL-AD scores remained consistent in sensitivity analyses using self-reported and proxy-reported scores. This implies that either perspective may be reliable for assessing QoL in PwMCI.

In site-specific analyses, significant effects on MMSE scores and QALY among PwMCI were observed at the SAS site in Spain, as well as improved medication adherence at the 6-month follow-up. These findings are exploratory and should be interpreted with caution, as they may reflect specific contextual factors, population characteristics, implementation nuances, or differences in local health care practices and caregiver support. Furthermore, the findings may be attributable to random chance. Future research should incorporate a priori effect-modifier analyses to formally test these associations and identify the specific factors, such as patient engagement, caregiver support, and local health care resources, that contributed to these positive outcomes.

The findings regarding PwMCI are consistent with existing evidence on the effectiveness of mobile apps for health promotion and disease management. A meta-analysis of 172 papers, primarily consisting of randomized trials, concluded that health-related apps provided a modest advantage in health outcomes compared with standard care [47]. A longitudinal study conducted in Ireland found no significant changes in the well-being of 12 PwD or their caregivers who used a smart health platform called Connected Health over a 12-month period. The Connected Health platform is a model of chronic care delivery that uses a portal to connect care providers and stakeholders, enabling a smooth and continuous flow of information. However, the results are not directly comparable because the Irish study lacked a control group, used a different QoL instrument (Dementia QoL), and had a smaller sample size consisting of patients with advanced dementia [48]. Another randomized clinical trial involving 390 persons aged 65 years and older evaluated the impact of an interactive website called ElderTree. The study found no significant improvements in QoL (Patient-Reported Outcomes Measurement Information System Global Health), independence (IADL), or related outcomes over a 12-month period [49].

We found no improvement in medication adherence with the SMART4MD app, which contrasts with a previous study by Li et al [50], who reported a significant improvement in medication adherence over 12 months using the Perx app. The Perx app included customized reminders and interactive games to incentivize medication adherence. However, the study population was younger and had health conditions other than cognitive decline. Additionally, users were required to upload a photo of their scheduled medication within 1 hour of receiving the reminder for verification [50]. Admittedly, SMART4MD did not include such interactive features. A scoping review also found that gamified apps may promote positive health behaviors, such as medication adherence [51], and may also drive app use [52]. Therefore, future mHealth apps may consider incorporating gamification strategies to support health behavior change, particularly medication adherence.

A direct comparison of our findings with previous studies of mHealth or eHealth interventions was not possible because of differences in the type of intervention, study design, outcome measures, and study duration. Another limiting factor is the limited evidence on the effectiveness of mHealth interventions designed for older adults with MCI or their informal caregivers. Therefore, it is difficult to draw definitive conclusions regarding the effectiveness of mHealth interventions for PwMCI and their informal caregivers based on the current evidence.

The high attrition rate in the current trial is unfortunately considered common in efficacy studies related to mHealth interventions [53]. However, to minimize attrition, SMART4MD trial investigators offered telephone interviews to participants who were unable to attend the 18-month follow-up in person. Consequently, 117 out of 650 (18%) follow-ups were conducted remotely by telephone. Most participants cited the COVID-19 pandemic as the reason for not participating in person at the 18-month follow-up. Although the pandemic may also have contributed to overall trial withdrawal, specific data on this reason for attrition were not collected at the time. Dropout was slightly higher in the intervention group (225/539, 41.7%) than in the control group (204/544, 37.5%), although the difference was not statistically significant. Assuming that dropout occurred randomly, the findings in Tables 2 and 3 provide unbiased estimates of health outcomes. However, we observed statistically significant differences in characteristics between dropouts and nondropouts (Tables S11 and S12 in Multimedia Appendix 2), indicating that missingness may not have been random, based on observed factors such as dyad age, decline in cognitive abilities, and increased caregiver burden. Previous studies have suggested using multiple imputation methods when missing data are assumed to be missing at random [54,55]. Sensitivity analyses based on multiple imputation of the primary outcome (QoL-AD) yielded significant effects (Table 5). Similarly, the exploratory results of the multiple imputation for QALY remained consistent with the base-case results, except that significant effects were observed at the 18-month follow-up for informal caregivers (nominal *P*=.02and dyads (nominal *P*=.01; see Tables S5 and S7 in Multimedia Appendix 2). Overall, we conclude that attrition is unlikely to have introduced substantial bias into the results. It is important to emphasize that significant differences in MMSE and ZBI scores between dropouts and nondropouts may suggest that the data were missing not at random. This implies that the likelihood of missing MMSE and ZBI data may have depended on baseline outcomes [56], although only 21 of 434 (4.8%) participants left the trial because of cognitive reasons.

As shown in the flowchart in Figure 1, most participants left the trial because they no longer wanted to continue participation. One possible reason for nonparticipation may have been difficulties in using the features of the SMART4MD app or dissatisfaction with the app relative to participants’ expectations [57]. However, a similar proportion of dyads withdrew from the control group, suggesting that the SMART4MD app itself may not have been the primary reason for attrition, but rather participation in the trial overall. Similar observations have been reported in other studies related to the SMART4MD project [22,58].

The small sample size in the medication adherence analysis reduced the likelihood of detecting statistically and clinically significant differences between groups. It is important to note that the number of participants recruited for the trial (1078 dyads) exceeded the required sample size for the primary outcome (QoL-AD), although it was lower than the required 1174 PwMCI needed to detect statistically and clinically significant differences in medication adherence [20]. At the 18-month follow-up, the sample included only 647 PwMCI for the main analysis and 418 PwMCI for the medication adherence analysis. The main reasons for further attrition in the medication adherence analysis were that participants reported not taking any medications or that the research nurse considered it impossible or inappropriate to count the medication.

In addition to the small sample size, the average number of times PwMCI launched the SMART4MD app was also low, indicating suboptimal utilization of the app. This may suggest that the SMART4MD app was not sufficiently user-friendly, making it challenging for PwMCI to use, as reported by Piculell et al [57]. A review of mHealth apps indicated a strong correlation between simple, easy-to-use, and interactive app features and their usability and acceptability [59]. If app functions are complex and PwMCI are unfamiliar with the app, this may even lead to distress [59]. A qualitative study based on individual semistructured interviews with 16 PwMCI from the SMART4MD trial found that participants did not perceive the app’s reminder feature as useful [57], which may have influenced their engagement with the app. This is further illustrated by the fact that only 195 of 312 (62.5%) PwMCI launched the app between the 6- and 12-month follow-ups, compared with 299 of 312 (95.8%) during the first 6 months. Participants may also have shown a lack of interest because they perceived themselves as too old to use a smartphone or tablet [57]. These findings suggest that participants may not have recognized the potential benefits of developing habits that could become useful as their condition progresses, underscoring the importance of educating participants about the benefits of mHealth interventions. As the nature of mHealth interventions differs from that of medications or surgical procedures, additional effort may be required to help participants appreciate the benefits associated with regular use of such interventions.

One of the inclusion criteria for the trial was that PwMCI had to have an MMSE score between 20 and 28. Although the use of an MMSE cutoff value of 28 is uncommon and associated with some limitations, it has been used in other studies [60]. Furthermore, previous research suggests that an MMSE cutoff score of 28 may be appropriate for identifying individuals with cognitive impairment, including those with mild dementia or MCI [23]. As PwMCI with an MMSE score of 28 had relatively higher cognitive abilities, we conducted sensitivity analyses using an MMSE cutoff of 26.

### Strengths and Limitations

The main strength of this study is that it was based on a large multicenter RCT conducted across different cultural and health care settings and included both PwMCI and their informal caregivers. Another strength is the use of multiple health outcome measures, which increased comparability with other studies. The main limitation is the high attrition rate, and the dropout analysis revealed significant differences between dropouts and nondropouts, indicating that the results should be interpreted with caution.

Another limitation is the lack of data on user behavior at the 18-month follow-up. Furthermore, there are concerns regarding the quality of the 12-month user behavior data because they only indicated whether the app had been launched. Detailed data on the duration of interactions with the app and the specific functions used were not collected; therefore, objective measures of actual app use were unavailable. Consequently, inferences regarding a causal relationship between app use or reminder exposure and changes in QoL among PwMCI and their caregivers should be interpreted with caution. The main analysis of this study was limited to examining the presence of app use. Therefore, the study did not fully capture the effect of reminder features for medication adherence and appointments on behavioral change. Low app-launch frequency may partly explain the absence of an effect on medication adherence, although the available usage data do not permit conclusions regarding exposure to specific reminder functions or actual engagement with medication-related features. In addition, it is possible that the app itself imposed an additional burden on PwMCI and their caregivers. The reminders may also have had nuisance effects by causing distraction among PwMCI. This interpretation is consistent with findings from the SMART4MD feasibility study, in which some participants reported feeling too old to use the technology and perceived the reminder functions as not useful [22]. Previous research suggests that mHealth apps for dementia are more likely to be adopted when their features are simple [61]. It is therefore possible that the functions of the SMART4MD app were not perceived as sufficiently simple or intuitive by PwMCI. Moreover, the 18-month follow-up period may not have been long enough to identify minimal clinically important differences, as PwMCI consistently reported minimal changes in their condition during this period. For example, for the MMSE, a follow-up period of at least 36 months has been suggested to observe clinically meaningful changes [62]. This observation is consistent with the existing literature [63]. The trial design focused on 2 frequently used medications, which may have introduced an upward bias in adherence reporting. PwMCI may exhibit higher adherence to well-established and frequently repeated routines than to more varied treatment regimens; consequently, our results may overestimate overall patient adherence.

Neither the researchers nor the participants were blinded to the intervention and control groups, which may have introduced bias into the outcomes. However, blinding may be less relevant in pragmatic trials that aim to replicate real-world practice as closely as possible [64]. Future research in this area could consider providing the control group with an app containing limited features to address this concern. Another limitation of the trial was the lack of detailed characterization of informal caregivers. For example, information on the primary caregiver’s relationship to the PwMCI, caregiving hours per day or week, and employment status was not documented. Furthermore, the effect of the SMART4MD app was not stratified by caregiver type (eg, spouse, adult child, or other), which could have provided insight into which groups benefited most from the intervention.

### Conclusions

The SMART4MD intervention was associated with a modest improvement in QoL among PwMCI, as measured by the composite QoL-AD score. Evidence of benefits among informal caregivers and dyads was less consistent and should be interpreted as exploratory. Further research is needed in this area, particularly studies with larger sample sizes, longer follow-up periods, and strategies to encourage app utilization. Future studies should also place greater emphasis on reducing dropout rates in mHealth programs.

## Appendix

Multimedia Appendix 1 CONSORT (Consolidated Standards of Reporting Trials) checklist.

Multimedia Appendix 2 Additional analyses.

## Abbreviations

AD: Alzheimer disease
BTH: Blekinge Institute of Technology
CONSORT: Consolidated Standards of Reporting Trials
CST: Consorci Sanitari de Terrassa
EVAS: European EQ-5D Visual Analogue Scale
IADL: Instrumental Activities of Daily Living
MCI: mild cognitive impairment
mHealth: mobile health
MMSE: Mini-Mental State Examination
PwMCI: people with mild cognitive impairment
QALY: quality-adjusted life years
QoL: quality of life
QoL-AD: Quality of Life in Alzheimer’s Disease
RCT: randomized controlled trial
SAS: Servicio Andaluz de Salud
SMART4MD: Support, Monitoring and Reminder Technology for Mild Dementia
ZBI: Zarit Burden Interview
