# Supplementary material for: Effects of the Digital App “Support, Monitoring and Reminder Technology for Mild Dementia” (SMART4MD) on People With Mild Cognitive Impairment and Their Informal Caregivers: 18-Month Multicenter Pragmatic Randomized Controlled Trial
Source: J Med Internet Res. 2026 Jul 24;28:e83123. doi: 10.2196/83123 (PMC13451873; doi:10.2196/83123)
Supplement: Supplementary file 2 [file jmir_v28i1e83123_app2.docx]

Multimedia Appendix 2

| **Table S1**  *Change in medication adherence (≥80% and ≤110%) between intervention and control group for at least one drug at 6-month, 12-month, and 18-month follow-up* | | | | | |
| --- | --- | --- | --- | --- | --- |
|  | Sample  Size | Intervention group | Control group | Absolute difference in probabilities | |
|  | (I/C) | Mean (SE) | Mean (SE) | Difference (95% CI) | *P-value* |
| Month 6 | 285/299 | 0.767 (0.02) | 0.710 (0.03) | 0.057 (-0.01 to 0.13) | .110 |
| Month 12 | 201/210 | 0.745 (0.03) | 0.753 (0.03) | -0.008 (-0.09 to 0.08) | .852 |
| Month 18 | 209/209 | 0.757 (0.03) | 0.742 (0.03) | 0.015 (-0.07 to 0.10) | .722 |
| Between group differences are computed using multilevel mixed effect logistic regression (melogit) and results are controlled for site. | | | | | |

| **Table S2**  *Interaction effects for the primary outcome QoL-AD for PwMCI* | | | | |
| --- | --- | --- | --- | --- |
| Scenarios | Reference group | Sample size | Changes in effect (QoL-AD) | *Interaction P-value* |
| Women | Male | 603 | 0.190 | .820 |
| Age >70 | Age ≤70 | 603 | -0.394 | .674 |
| MMSE > 26 | MMSE ≤26 | 603 | -1.246 | .123 |
| Abbreviations: MMSE: mini-mental state examination; PwMCI: persons with mild cognitive impairment; QoL-AD: quality of life in Alzheimer’s disease scale. | | | | |

| **Table S3**  *Demographic characteristics of PwMCI and their informal caregivers at baseline and 18-month follow-up* | | | | | | | | | | | | | |
| --- | --- | --- | --- | --- | --- | --- | --- | --- | --- | --- | --- | --- | --- |
| PwMCI | | | | | | | Informal Caregiver | | | | | | |
|  | Baseline | | | 18-month follow-up | | | Baseline | | | 18-month follow-up | | | |
|  | Intervention (n=173) | Control (n=172) | *P* value | Intervention (n=131) | Control (n=141) | *P* value | Intervention (n=173) | Control (n=172) | *P* value | Intervention (n=127) | Control (n=137) | *P* value |  |
| Age^a^, years | 75 (7.2) | 74 (7.2) | .47 | 75 (6.9) | 76 (6.7) | .65 | 62 (15.0) | 63 (14.3) | .33 | 65 (13.99) | 65 (14.27) | .87 |  |
| Sex, n (%) | | | | | | | | | | | | | |
| Male | 241 (45) | 265 (49) | .18 | 156 (50) | 172 (51) | .73 | 166 (31) | 178 (33) | .48 | 82 (28) | 102 (32) | .26 |  |
| Female | 296 (55) | 276 (51) |  | 156 (50) | 163 (49) |  | 371 (69) | 363 (67) |  | 214 (72) | 218 (68) |  |  |
| Education ^b^, n (%) | | | | | | | | | | | | | |
| Elementary education | 313 (59) | 333 (62) | .17 | 179 (57) | 200 (60) | .07 | 175 (33) | 201 (38) | .09 | 105 (36) | 117 (37) | .16 |  |
| Secondary education | 124 (23) | 100 (18) |  | 74 (24) | 57 (17) |  | 186 (36) | 156 (29) |  | 106 (37) | 95 (30) |  |  |
| Higher education | 97 (18) | 106 (20) |  | 58 (19) | 77 (23) |  | 161 (31) | 173 (33) |  | 77 (27) | 102 (33) |  |  |
| Civil status, n (%) | | | | | | | | | | | | | |
| Single | 181 (34) | 159 (29) | .13 | 99 (32) | 87 (26) | .11 | 105 (20) | 95 (18) | .39 | 52 (18) | 60 (19) | .69 |  |
| Married/living together | 356 (66) | 381 (71) |  | 213 (68) | 248 (74) |  | 430 (80) | 445 (82) |  | 244 (82) | 259 (81) |  |  |
| Living arrangements, n (%) | | | | | | | | | | | | | |
| Single | 117 (22) | 102 (19) | .16 | 73 (23) | 50 (15) | .007 | 52 (10) | 43 (8) | .65 | 27 (9) | 19 (6) | .038 |  |
| Spouse/Common law partner | 327 (61) | 356 (66) |  | 205 (66) | 240 (72) |  | 357 (67) | 378 (70) |  | 217 (73) | 246 (77) |  |  |
| Children | 56 (11) | 41 (8) |  | 20 (6) | 15 (4) |  | 53 (10) | 48 (9) |  | 27 (9) | 16 (5) |  |  |
| Other | 32 (6) | 39 (7) |  | 14 (5) | 29 (9) |  | 72 (13) | 72 (13) |  | 25 (9) | 40 (12) |  |  |
| Composite QoL-AD ^a, d^ | 35.69 (5.94) | 35.53 (6.17) | .66 | 36.58 (5.15) | 35.98 (5.11) | .15 |  |  |  |  |  |  |  |
| EQ-5D-3L index scores ^a, e^ (EVAS) | 0.770 (0.19) | 0.772 (0.21) | .87 | 0.761 (0.20) | 0.756 (0.20) | .73 | 0.801 (0.18) | 0.786 (0.21) | .21 | 0.800 (0.18) | 0.770 (0.20) | .05 |  |
| MMSE score ^a, f^ | 25.39 (2.51) | 25.49 (2.40) | .48 | 26.00 (4.24) | 25.62 (3.90) | .24 |  |  |  |  |  |  |  |
| IADL score ^a, g^ | 6.70 (1.66) | 6.65 (1.70) | .61 | 6.06 (1.98) | 6.06 (2.06) | .996 |  |  |  |  |  |  |  |
| ZBI scores ^a, h^ |  |  |  |  |  |  | 6.76 (7.43) | 6.86 (7.88) | .82 | 6.45 (7.83) | 6.90 (7.50) | .47 |  |
| Abbreviations: IADL: Instrumental activities of daily living; MMSE: mini-mental state examination; PwMCI: persons with mild cognitive impairment; QoL-AD: quality of life in Alzheimer’s disease scale; ZBI: Zarit Caregiver Burden Inventory.  Note: a: Mean (standard deviation).  b: sample size of informal caregivers at 18-month follow-up (Intervention: 288; Control: 314).  c: sample size of informal caregivers at 18-month follow-up (Control: 319).  d: sample size at baseline (Intervention: 532; Control: 536); at18-month follow-up (Intervention: 289; Control: 314).  e: sample size of PwMCI at baseline (Intervention: 537); sample size of PwMCI at18-month follow-up (Intervention: 311; Control: 334); sample size of informal caregiver at baseline (Intervention: 535; Control: 535); sample size of informal caregiver at18-month follow-up (Intervention: 296; Control: 320).  f: sample size of PwMCI at18-month follow-up (Intervention: 310).  g: sample size of PwMCI at baseline (Intervention: 471; Control: 487); sample size of PwMCI at18-month follow-up (Intervention: 292; Control: 312).  h: sample size of informal caregiver at baseline (Intervention: 536; Control: 538); sample size of informal caregiver at18-month follow-up (Intervention: 292; Control: 318); | | | | | | | | | | | | | |

| **Table S4**  *Baseline to 18-month intragroup differences in effect measures in intervention and control groups* | | | | | | | | |
| --- | --- | --- | --- | --- | --- | --- | --- | --- |
|  | Intervention Group | | | | Control Group | | | |
|  | Sample size | Follow-up | Baseline | Difference (*P-value*) | Sample size | Follow-up | Baseline | Difference (*P-value*) |
| **PwMCI** | | | | | | | | |
| Composite QoL-AD ^a^ | 287 | 36.613 (5.15) | 36.254 (6.17) | 0.359 (.176) | 310 | 35.952 (5.14) | 36.323 (6.19) | -0.371 (.089) |
| EQ-5D-3L index scores ^a^ (EVAS) | 311 | 0.761 (0.20) | 0.781 (0.19) | -0.020 (.077) | 334 | 0.756 (0.20) | 0.784 (0.20) | -0.028(.006) |
| MMSE score ^a^ | 310 | 25.997 (4.24) | 26.026 (2.11) | -0.029 (.876) | 335 | 25.621 (3.90) | 25.896 (2.20) | -0.275 (.081) |
| IADL score ^a^ | 265 | 6.155 (1.95) | 7.038 (1.41) | -0.883 (<.001) | 283 | 6.152 (2.02) | 6.859 (1.63) | -0.707 (<.001) |
| **Informal Caregiver** | | | | | | | | |
| EQ-5D-3L index scores ^a^ (EVAS) | 295 | 0.800 (0.18) | 0.809 (0.17) | -0.009 (.429) | 316 | 0.769 (0.20) | 0.784 (0.21) | -0.015 (.237) |
| ZBI scores ^a^ | 292 | 6.445 (7.83) | 6.034 (7.47) | 0.411 (.311) | 316 | 6.937 (7.51) | 6.272 (7.47) | 0.665 (.143) |
| **Dyads (PwMCI plus Informal Caregiver)** | | | | | | | | |
| EQ-5D-3L index scores ^a^ (EVAS) | 294 | 1.566 (0.28) | 1.594 (0.28) | -0.028 (.109) | 313 | 1.523 (0.29) | 1.571 (0.30) | -0.048 (.006) |
| Abbreviations: IADL: Instrumental activities of daily living; MMSE: mini-mental state examination; PwMCI: persons with mild cognitive impairment; QoL-AD: quality of life in Alzheimer’s disease scale; ZBI: Zarit Caregiver Burden Inventory. Intra-group (within group) differences are computed using paired t-test. Note: a: Mean (standard deviation); b: P-values for exploratory outcomes are nominal and have not been adjusted for multiple comparisons. | | | | | | | | |

| **Table S5**  *Sensitivity analysis for total accumulated QALY based on EVAS for PwMCI* | | | | | |
| --- | --- | --- | --- | --- | --- |
|  | Sample  Size | Intervention group | Control group | Difference |  |
|  | (I/C)^a^ | Mean (SE)^b^ | Mean (SE)^b^ | Mean (95% CI) | *P-value^c^* |
| **Base case** |  |  |  |  |  |
| Month 6 | 395/442 | 0.423 (0.006) | 0.388 (0.005) | 0.035 (0.02 to 0.05) | <.001 |
| Month 12 | 340/383 | 0.365 (0.006) | 0.380 (0.006) | -0.015 (−0.03 to 0.00) | .079 |
| Month 18 | 298/334 | 0.365 (0.006) | 0.353 (0.006) | 0.012 (−0.01 to 0.03) | .166 |
| **Imputed QALY** |  |  |  |  |  |
| Month 6 | 532/535 | 0.420 (0.004) | 0.384 (0.004) | 0.036 (0.02 to 0.05) | <.001 |
| Month 12 | 532/534 | 0.363 (0.004) | 0.375 (0.004) | -0.011 (-0.02 to 0.001) | .077 |
| Month 18 | 529/533 | 0.362 (0.004) | 0.352 (0.004) | 0.010 (-0.003 to 0.02) | .127 |
| **High app use >125** |  |  |  |  |  |
| Month 6 | 174/442 | 0.435 (0.01) | 0.389 (0.01) | 0.046 (0.03 to 0.07) | <.001 |
| Month 12 | 173/383 | 0.363 (0.01) | 0.380 (0.01) | -0.017 (-0.04 to 0.003) | .098 |
| Month 18 | 175/334 | 0.366 (0.01) | 0.353 (0.01) | 0.013 (-0.008 to 0.03) | .219 |
| **Low app use≤125** |  |  |  |  |  |
| Month 6 | 178/442 | 0.414 (0.01) | 0.388 (0.01) | 0.025 (0.006 to 0.04) | .009 |
| Month 12 | 164/383 | 0.375 (0.01) | 0.380 (0.01) | -0.005 (-0.03 to 0.01) | .620 |
| Month 18 | 117/334 | 0.375 (0.01) | 0.353 (0.01) | 0.022 (0.0003 to 0.04) | .047 |
| Abbreviations: PwMCI: persons with mild cognitive impairment.  a: (Intervention group/Control group); b: Mean (Standard Error); c: P-values for exploratory outcomes are nominal and have not been adjusted for multiple comparisons.  *Note:* Incremental effect with positive value represent improved outcomes.; *Multiple imputations for missing QALY:* We used multiple imputations to impute missing information on EQ-5D-3L index score for both groups [1], generating twenty different data sets. The baseline variables used in the models are age, sex, education, living arrangements, EQ-5D-3L index score, QoL-AD score of PwMCI, MMSE score, ZBI score, and intervention/control group. Then we analyzed the multiple datasets and computed pooled estimates following the methods described by Rubin [2]. | | | | | |

| **Table S6**  *Interaction terms analysis for total accumulated QALY based on EVAS* | | | | |
| --- | --- | --- | --- | --- |
| Scenarios | Reference group | Sample size | Changes in effect (QALY) | *Interaction P-value* |
| **PwMCI** | | | |  |
| Women | Male | 610 | 0.027 | .528 |
| Age >70 | Age ≤70 | 610 | -0.081 | .107 |
| MMSE > 26 | MMSE ≤26 | 610 | -0.068 | .122 |
| **Informal Caregiver** | | | |  |
| Women | Male | 557 | 0.040 | .368 |
| Age >70 | Age ≤70 | 557 | 0.035 | .403 |
| ZBI ≤31 (Some burden) | ZBI >31 (No burden) | 557 | -0.071 | .331 |
| Abbreviations: MMSE: mini-mental state examination; PwMCI: persons with mild cognitive impairment; QoL-AD: quality of life in Alzheimer’s disease scale. | | | | |

| **Table S7**  *Sensitivity analysis for total accumulated QALY based on EVAS for informal carer and dyad* | | | | | |
| --- | --- | --- | --- | --- | --- |
|  | Sample  Size | Intervention group | Control group | Difference |  |
|  | (I/C)^a^ | Mean (SE)^b^ | Mean (SE)^b^ | Mean (95% CI) | *P-value^c^* |
| **Informal Caregiver** |  |  |  |  |  |
| **Base case** |  |  |  |  |  |
| Month 6 | 375/422 | 0.448 (0.006) | 0.398 (0.005) | 0.050 (0.04 to 0.07) | <.001 |
| Month 12 | 314/354 | 0.388 (0.006) | 0.397 (0.006) | -0.009 (-0.03 to 0.01) | 0.265 |
| Month 18 | 279/303 | 0.385 (0.006) | 0.376 (0.006) | 0.009 (-0.01 to 0.03) | 0.315 |
| **Imputed QALY** |  |  |  |  |  |
| Month 6 | 511/513 | 0.443 (0.004) | 0.399 (0.004) | 0.043 (0.03 to 0.05) | <.001 |
| Month 12 | 507/516 | 0.388 (0.004) | 0.397 (0.004) | -0.010 (-0.02 to 0.002) | .111 |
| Month 18 | 503/513 | 0.385 (0.004) | 0.370 (0.004) | 0.014 (0.003 to 0.03) | .016 |
| **High app use >125** |  |  |  |  |  |
| Month 6 | 165/422 | 0.450 (0.008) | 0.399 (0.005) | 0.051 (0.03 to 0.07) | <.001 |
| Month 12 | 161/354 | 0.381 (0.008) | 0.397 (0.006) | -0.016 (-0.04 to 0.003) | .102 |
| Month 18 | 166/303 | 0.382 (0.008) | 0.376 (0.006) | 0.006 (-0.01 to 0.03) | .582 |
| **Low app use≤125** |  |  |  |  |  |
| Month 6 | 165/422 | 0.447 (0.008) | 0.398 (0.005) | 0.049 (0.03 to 0.07) | <.001 |
| Month 12 | 153/354 | 0.395 (0.009) | 0.396 (0.006) | -0.002 (-0.02 to 0.02) | .881 |
| Month 18 | 110/303 | 0.390 (0.010) | 0.376 (0.006) | 0.014 (-0.008 to 0.04) | .215 |
| **Dyads (PwMCI plus Informal Caregiver)** |  |  |  |  |  |
| **Base case** |  |  |  |  |  |
| Month 6 | 372/420 | 0.874 (0.009) | 0.789 (0.009) | 0.085 (0.06 to 0.11) | <.001 |
| Month 12 | 312/350 | 0.759 (0.010) | 0.781 (0.009) | -0.022 (-0.05 to 0.005) | .115 |
| Month 18 | 276/300 | 0.758 (0.011) | 0.739 (0.010) | 0.018 (-0.01 to 0.05) | .210 |
| **Imputed QALY** |  |  |  |  |  |
| Month 6 | 510/511 | 0.863 (0.007) | 0.785 (0.007) | 0.079 (0.06 to 0.10) | <.001 |
| Month 12 | 507/514 | 0.753 (0.007) | 0.773 (0.007) | -0.020 (-0.04 to -0.0005) | .045 |
| Month 18 | 503/511 | 0.748 (0.007) | 0.723 (0.007) | 0.025 (0.005 to 0.04) | .014 |
| Abbreviations: QALY: quality-adjusted life years.  a: (Intervention group/Control group); b: Mean (Standard Error); c: P-values for exploratory outcomes are nominal and have not been adjusted for multiple comparisons.  *Note:* Incremental effect with positive value represent improved outcomes.; *Multiple imputations for missing QALY:* We used multiple imputations to impute missing information on EQ-5D-3L index score for both groups [1], generating twenty different data sets. The baseline variables used in the models are age, sex, education, living arrangements, EQ-5D-3L index score, QoL-AD score of PwMCI, MMSE score, ZBI score, and intervention/control group. Then we analyzed the multiple datasets and computed pooled estimates following the methods described by Rubin [2] | | | | | |

| **Table S8**  *Inter-group differences in health outcomes of PwMCI by sites* | | | | | | | | | | | | | | | |
| --- | --- | --- | --- | --- | --- | --- | --- | --- | --- | --- | --- | --- | --- | --- | --- |
|  | BTH | | | | | CST | | | | | SAS | | | | |
|  | Sample size | Intervention group | Control gruop | Difference | | Sample size | Intervention group | Control gruop | Difference | | Sample size | Intervention group | Control gruop | Difference | |
|  | I/C^a^ | Mean (SE)^b^ | Mean (SE)^b^ | Mean (95% CI) | *P-value* ^b^ | I/C^a^ | Mean (SE) ^b^ | Mean (SE) ^b^ | Mean (95% CI) | *P-value* ^b^ | I/C^a^ | Mean (SE) ^b^ | Mean (SE) ^b^ | Mean (95% CI) | *P-value* ^b^ |
| **PwMCI** | | | | | | | | | | | | | | |  |
| **Composite QoL-AD** |  |  |  |  |  |  |  |  |  |  |  |  |  |  |  |
| Baseline | 169/167 | 40.27 (0.38) | 40.25 (0.38) | 0.022 (-1.03 to 1.07) | .967 | 166/166 | 33.95 (0.33) | 33.11 (0.33) | 0.844 (-0.07 to 1.76) | .071 | 181/184 | 33.10 (0.39) | 33.57 (0.39) | -0.471 (-1.56 to 0.62) | .396 |
| Month 6 | 133/154 | 39.44 (0.40) | 39.04 (0.39) | 0.410 (-0.68 to 1.50) | .460 | 116/130 | 33.96 (0.36) | 33.19 (0.35) | 0.770 (-0.22 to 1.76) | .126 | 126/142 | 35.76 (0.43) | 34.77 (0.41) | 0.989 (-0.18 to 2.15) | .096 |
| Month 12 | 121/129 | 38.76 (0.40) | 38.89 (0.40) | -0.123 (-1.23 to 0.99) | .829 | 53/68 | 34.42 (0.45) | 32.79 (0.41) | 1.62 (0.43 to 2.81) | .008 | 85/97 | 35.36 (0.46) | 34.85 (0.45) | 0.513 (-0.75 to 1.78) | .426 |
| Month 18 | 120/134 | 38.67 (0.40) | 38.59 (0.40) | 0.082 (-1.03 to 1.19) | .884 | 68/75 | 34.21 (0.41) | 33.25 (0.40) | 0.969 (-0.16 to 2.10) | .093 | 101/105 | 35.57 (0.45) | 34.40 (0.44) | 1.170 (-0.06 to 2.40) | .062 |
| **EQ-5D-3L index scores (EVAS)** |  |  |  |  |  |  |  |  |  |  |  |  |  |  |  |
| Baseline | 173/172 | 0.790 (0.01) | 0.796 (0.01) | -0.006 (-0.04 to 0.03) | .736 | 173/170 | 0.750 (0.02) | 0.734 (0.02) | 0.016 (-0.03 to 0.06) | .487 | 190/196 | 0.771 (0.01) | 0.787 (0.01) | -0.015 (-0.06 to 0.03) | .468 |
| Month 6 | 138/158 | 0.766 (0.01) | 0.773 (0.01) | -0.007 (-0.05 to 0.03) | .733 | 125/133 | 0.733 (0.02) | 0.723 (0.02) | 0.010 (-0.04 to 0.06) | .687 | 132/150 | 0.785 (0.02) | 0.757 (0.02) | 0.029 (-0.02 to 0.07) | .210 |
| Month 12 | 134/147 | 0.766 (0.01) | 0.777 (0.01) | -0.011 (-0.05 to 0.03)0.586 | .586 | 101/117 | 0.729 (0.02) | 0.712 (0.02) | 0.016 (-0.04 to 0.07) | .543 | 115/132 | 0.776 (0.02) | 0.778 (0.02) | -0.002 (-0.05 to 0.04) | .933 |
| Month 18 | 130/141 | 0.780 (0.01) | 0.774 (0.01) | 0.006 (-0.03 to 0.05) | .761 | 74/79 | 0.735 (0.02) | 0.708 (0.02) | 0.027 (-0.03 to 0.09) | .368 | 107/114 | 0.752 (0.02) | 0.768 (0.02) | -0.016 (-0.06 to 0.03) | .510 |
| **Total accumulated QALY (EVAS)** |  |  |  |  |  |  |  |  |  |  |  |  |  |  |  |
| Month 6 | 139/158 | 0.380 (0.008) | 0.386 (0.008) | -0.006 (-0.03 to 0.02) | .567 | 124/133 | 0.397 (0.01) | 0.378 (0.01) | 0.020 (-0.01 to 0.05) | .175 | 132/151 | 0.492 (0.01) | 0.399 (0.01) | 0.093 (0.06 to 0.12) | <.001 |
| Month 12 | 130/146 | 0.381 (0.008) | 0.400 (0.008) | -0.019 (-0.04 to 0.003) | .093 | 99/110 | 0.359 (0.01) | 0.358 (0.01) | 0.001 (-0.03 to 0.03) | .942 | 111/127 | 0.348 (0.01) | 0.374 (0.01) | -0.026 (-0.06 to 0.01) | .107 |
| Month 18 | 130/145 | 0.386 (0.008) | 0.376 (0.008) | 0.010 (-0.01 to 0.03) | .370 | 69/74 | 0.380 (0.01) | 0.333 (0.01) | 0.047 (0.01 to 0.08) | .008 | 99/115 | 0.325 (0.01) | 0.339 (0.01) | -0.014 (-0.05 to 0.02) | .404 |
| **Total accumulated QALY (SWE)** |  |  |  |  |  |  |  |  |  |  |  |  |  |  |  |
| Month 6 | 139/158 | 0.428 (0.006) | 0.432 (0.006) | -0.004 (-0.02 to 0.01) | .636 |  |  |  |  |  |  |  |  |  |  |
| Month 12 | 130/146 | 0.431 (0.007) | 0.448 (0.006) | -0.017 (-0.03 to 0.001) | .062 |  |  |  |  |  |  |  |  |  |  |
| Month 18 | 130/145 | 0.438 (0.007) | 0.423 (0.006) | 0.015 (-0.003 to 0.03) | .099 |  |  |  |  |  |  |  |  |  |  |
| **Total accumulated QALY (Spanish)** |  |  |  |  |  |  |  |  |  |  |  |  |  |  |  |
| Month 6 |  |  |  |  |  | 124/134 | 0.401 (0.01) | 0.380 (0.01) | 0.021 (-0.01 to 0.06) | .243 | 132/152 | 0.503 (0.01) | 0.405 (0.01) | 0.098 (0.06 to 0.13) | <.001 |
| Month 12 |  |  |  |  |  | 99/111 | 0.363 (0.01) | 0.358 (0.01) | 0.004 (-0.03 to 0.04) | .830 | 111/127 | 0.359 (0.01) | 0.381 (0.01) | -0.022 (-0.06 to 0.01) | .231 |
| Month 18 |  |  |  |  |  | 69/74 | 0.384 (0.02) | 0.334 (0.01) | 0.051 (0.01 to 0.09) | .018 | 99/115 | 0.334 (0.01) | 0.346 (0.01) | -0.012 (-0.05 to 0.02) | .517 |
| **MMSE score** |  |  |  |  |  |  |  |  |  |  |  |  |  |  |  |
| Baseline | 173/172 | 26.53 (0.18) | 26.78 (0.18) | -0.253 (-0.75 to 0.25) | .321 | 174/170 | 23.80 (0.29) | 24.42 (0.30) | -0.619 (-1.44 to 0.20) | .139 | 190/196 | 25.79 (0.22) | 25.34 (0.22) | 0.453 (-0.15 to 1.06) | .143 |
| Month 6 | 138/158 | 27.63 (0.19) | 27.40 (0.19) | 0.232 (-0.29 to 0.76) | .386 | 124/132 | 22.85 (0.32) | 23.35 (0.32) | -0.503 (-1.39 to 0.39) | .267 | 132/150 | 25.54 (0.25) | 25.35 (0.24) | 0.197 (-0.47 to 0.87) | .563 |
| Month 12 | 134/147 | 27.58 (0.20) | 27.49 (0.19) | 0.093 (-0.44 to 0.63) | .732 | 101/117 | 22.72 (0.34) | 24.00 (0.33) | -1.28 (-2.20 to -0.35) | .007 | 117/134 | 24.68 (0.26) | 24.64 (0.24) | 0.046 (-0.65 to 0.74) | .897 |
| Month 18 | 130/141 | 27.59 (0.20) | 27.25 (0.19) | 0.339 (-0.20 to 0.88) | .217 | 73/80 | 22.40 (0.37) | 23.38 (0.36) | -0.986 (-2.01 to 003) | .058 | 107/114 | 25.50 (0.26) | 24.64 (0.25) | 0.868 (0.15 to 1.58) | .018 |
| **IADL score** |  |  |  |  |  |  |  |  |  |  |  |  |  |  |  |
| Baseline | 151/152 | 7.41 (0.11) | 7.35 (0.11) | 0.059 (-0.24 to 0.36) | .701 | 144/148 | 6.31 (0.16) | 6.16 (0.16) | 0.147 (-0.30 to 0.59) | .520 | 176/184 | 6.33 (0.15) | 6.34 (0.15) | -0.005 (-0.42 to 0.41) | .981 |
| Month 6 | 129/145 | 6.88 (0.12) | 7.14 (0.11) | -0.256 (-0.57 to 0.06) | .110 | 110/116 | 5.51 (0.17) | 5.36 (0.17) | 0.151 (-0.32 to 0.63) | .532 | 124/144 | 6.02 (0.16) | 5.93 (0.16) | 0.087 (-0.35 to 0.53) | .697 |
| Month 12 | 124/100 | 6.69 (0.12) | 6.84 (0.13) | -0.151 (-0.49 to 0.19) | .382 | 91/100 | 5.42 (0.18) | 5.27 (0.18) | 0.150 (-0.34 to 0.64) | .553 | 115/130 | 5.75 (0.16) | 6.03 (0.16) | -0.282 (-0.73 to 0.17) | .218 |
| Month 18 | 117/127 | 6.56 (0.12) | 6.74 (0.12) | -0.177 (-0.50 to 0.15) | .289 | 69/73 | 5.10 (0.19) | 4.96 (0.19) | 0.142 (-0.39 to 0.67) | .599 | 106/112 | 5.67 (0.17) | 5.75 (0.16) | -0.081 (-0.54 to 0.38) | .731 |
| Abbreviations: BTH: Blekinge Institute of Technology, Karlskrona, Sweden; CST: Consorci Sanitaria de Terrassa, Barcelona, Spain; IADL: Lawton instrumental activities of daily living; MMSE: mini-mental state examination; PwMCI: persons with mild cognitive impairment; QoL-AD: quality of life in Alzheimer disease; QALY: quality adjusted life years; SAS: Service Andaluz de Salud, Málaga, Spain.  a: (Intervention group/Control group); b: Mean (Standard Error); c: P-values for exploratory outcomes are nominal and have not been adjusted for multiple comparisons. | | | | | | | | | | | | | | | |

| **Table S9**  *Inter-group differences in health outcomes of informal caregivers and Dyads by sites* | | | | | | | | | | | | | | | |
| --- | --- | --- | --- | --- | --- | --- | --- | --- | --- | --- | --- | --- | --- | --- | --- |
|  | BTH | | | | | CST | | | | | SAS | | | | |
|  | Sample size | Intervention group | Control gruop | Difference | | Sample size | Intervention group | Control gruop | Difference | | Sample size | Intervention group | Control gruop | Difference | |
|  | I/C^a^ | Mean (SE)^b^ | Mean (SE)^b^ | Mean (95% CI) | *P-value*^c^ | I/C^a^ | Mean (SE)^b^ | Mean (SE)^b^ | Mean (95% CI) | *P-value*^c^ | I/C^a^ | Mean (SE)^b^ | Mean (SE)^b^ | Mean (95% CI) | *P-value*^c^ |
| **Informal Caregiver** | | | | | | | | | | | | | | | |
| **EQ-5D-3L index scores (EVAS)** |  |  |  |  |  |  |  |  |  |  |  |  |  |  |  |
| Baseline | 169/167 | 0.808 (0.01) | 0.788 (0.01) | 0.021 (-0.02 to 0.06) | .296 | 168/168 | 0.773 (0.02) | 0.746 (0.02) | 0.027 (-0.02 to 0.07) | .237 | 182/183 | 0.817 (0.01) | 0.823 (0.01) | -0.006 (-0.04 to 0.03) | .783 |
| Month 6 | 134/154 | 0.794 (0.01) | 0.787 (0.01) | 0.006 (-0.03 to 0.05) | .758 | 117/132 | 0.811 (0.02) | 0.775 (0.02) | 0.035 (-0.02 to 0.09) | .171 | 125/141 | 0.842 (0.02) | 0.821 (0.02) | 0.021 (-0.02 to 0.07) | .354 |
| Month 12 | 129/141 | 0.763 (0.02) | 0.770 (0.01) | -0.006 (-0.05 to 0.03) | .763 | 93/108 | 0.767 (0.02) | 0.737 (0.02) | 0.030 (-0.03 to 0.08) | .294 | 109/122 | 0.844 (0.02) | 0.844 (0.02) | 0.001 (-0.05 to 0.05) | .981 |
| Month 18 | 127/137 | 0.786 (0.02) | 0.755 (0.01) | 0.031 (-0.01 to 0.07) | .144 | 68/78 | 0.762 (0.02) | 0.717 (0.02) | 0.045 (-0.02 to 0.11) | .155 | 101/105 | 0.839 (0.02) | 0.825 (0.02) | 0.014 (-0.03 to 0.06) | .578 |
| **Total accumulated QALY (EVAS)** |  |  |  |  |  |  |  |  |  |  |  |  |  |  |  |
| Month 6 | 134/153 | 0.392 (0.01) | 0.385 (0.01) | 0.007 (-0.01 to 0.03) | .507 | 117/132 | 0.435 (0.01) | 0.389 (0.01) | 0.046 (0.02 to 0.07) | .001 | 124/137 | 0.523  (0.01) | 0.422 (0.01) | 0.101 (0.07 to 0.13) | <.001 |
| Month 12 | 124/139 | 0.386 (0.01) | 0.400 (0.01) | -0.015 (-0.04 to 0.01) | .198 | 88/100 | 0.396 (0.01) | 0.381 (0.01) | 0.014 (-0.02 to 0.04) | .343 | 102/115 | 0.381 (0.01) | 0.406 (0.01) | -0.024 (-0.05 to 0.01) | .120 |
| Month 18 | 125/135 | 0.388 (0.01) | 0.377 (0.01) | 0.011 (-0.01 to 0.03) | .323 | 63/67 | 0.399 (0.01) | 0.370 (0.01) | 0.030 (-0.01 to 0.06) | .095 | 91/101 | 0.363 (0.01) | 0.379 (0.01) | -0.016 (-0.05 to 0.02) | .323 |
| **Total accumulated QALY (SWE)** |  |  |  |  |  |  |  |  |  |  |  |  |  |  |  |
| Month 6 | 134/153 | 0.435 (0.006) | 0.430 (0.006) | 0.005 (-0.01 to 0.02) | .540 |  |  |  |  |  |  |  |  |  |  |
| Month 12 | 124/139 | 0.435 (0.006) | 0.447 (0.006) | -0.012 (-0.03 to 0.004) | .145 |  |  |  |  |  |  |  |  |  |  |
| Month 18 | 125/135 | 0.441 (0.006) | 0.427 (0.006) | 0.013 (-0.003 to 0.03) | .114 |  |  |  |  |  |  |  |  |  |  |
| **Total accumulated QALY (Spanish)** |  |  |  |  |  |  |  |  |  |  |  |  |  |  |  |
| Month 6 |  |  |  |  |  | 117/132 | 0.449 (0.01) | 0.398 (0.01) | 0.051 (0.02 to 0.08) | .002 | 124/137 | 0.538 (0.01) | 0.433 (0.01) | 0.105 (0.07 to 0.14) | <.001 |
| Month 12 |  |  |  |  |  | 88/100 | 0.409 (0.01) | 0.392 (0.01) | 0.017 (-0.02 to 0.05) | .347 | 102/115 | 0.391 (0.01) | 0.416 (0.01) | -0.025 (-0.06 to 0.01) | .140 |
| Month 18 |  |  |  |  |  | 63/67 | 0.416 (0.02) | 0.376 (0.01) | 0.040 (-0.001 to 0.08) | .058 | 91/101 | 0.374 (0.01) | 0.392 (0.01) | -0.018 (-0.05 to 0.02) | .319 |
| **ZBI** |  |  |  |  |  |  |  |  |  |  |  |  |  |  |  |
| Baseline | 169/167 | 4.46 (0.48) | 4.93 (0.48) | -0.469 (-1.81 to 0.87) | .492 | 168/168 | 9.04 (0.63) | 9.08 (0.63) | -0.034 (-1.77 to 1.70) | .970 | 183/186 | 6.80 (0.57) | 6.86 (0.56) | -0.059 (-1.62 to 1.51) | .941 |
| Month 6 | 134/154 | 4.70 (0.51) | 5.04 (0.49) | -0.340 (-1.74 to 1.06) | .633 | 118/132 | 9.39 (0.69) | 9.46 (0.67) | -0.072 (-1.96 to 1.82) | .940 | 126/141 | 6.61 (0.65) | 6.34 (0.62) | 0.275 (-1.49 to 2.04) | .760 |
| Month 12 | 129/142 | 3.77 (0.52) | 4.49 (0.51) | -0.714 (-2.13 to 0.71) | .325 | 93/107 | 9.27 (0.74) | 10.07 (0.71) | -0.802 (-2.81 to 1.20) | .433 | 110/121 | 7.41 (0.68) | 6.77 (0.66) | 0.642 (-1.21 to 2.50) | .497 |
| Month 18 | 126/137 | 4.11 (0.52) | 5.26 (0.51) | -1.14 (-2.58 to 0.29) | .117 | 68/78 | 10.45 (0.81) | 10.92 (0.77) | -0.470 (-2.66 to 1.72) | .675 | 98/103 | 7.63 (0.71) | 7.50 (0.69) | 0.125 (-1.82 to 2.07) | .900 |
| **Dyad (PwMCI plus informal caregiver)** | | | | | | | | | | | | | | | |
| **Total accumulated QALY (EVAS)** |  |  |  |  |  |  |  |  |  |  |  |  |  |  |  |
| Month 6 | 134/153 | 0.775 (0.01) | 0.770 (0.01) | 0.005 (-0.03 to 0.04) | .767 | 114/130 | 0.839 (0.02) | 0.771 (0.02) | 0.068 (0.02 to 0.11) | .003 | 124/137 | 1.01 (0.02) | 0.826 (0.02) | 0.187 (0.14 to 0.24) | <.001 |
| Month 12 | 124/139 | 0.769 (0.01) | 0.802 (0.01) | -0.033 (-0.07 to 0.001) | .055 | 88/98 | 0.758 (0.02) | 0.744 (0.02) | 0.015 (-0.04 to 0.07) | .567 | 100/113 | 0.742 (0.02) | 0.784 (0.02) | -0.042 (-0.10 to 0.01) | .124 |
| Month 18 | 124/135 | 0.780 (0.01) | 0.763 (0.01) | 0.017 (-0.02 to 0.05) | .325 | 63/64 | 0.785 (0.02) | 0.718 (0.02) | 0.067 (0.01 to 0.13) | .027 | 89/101 | 0.701 (0.02) | 0.723 (0.02) | -0.022 (-0.08 to 0.03) | .440 |
| **Total accumulated QALY (SWE)** |  |  |  |  |  |  |  |  |  |  |  |  |  |  |  |
| Month 6 | 134/153 | 0.864 (0.010) | 0.862 (0.009) | 0.003 (-0.02 to 0.03) | .842 |  |  |  |  |  |  |  |  |  |  |
| Month 12 | 124/139 | 0.868* (0.010) | 0.898 (0.010) | -0.030 (-0.06 to -0.002) | .033 |  |  |  |  |  |  |  |  |  |  |
| Month 18 | 124/135 | 0.885 (0.010) | 0.863 (0.010) | 0.022 (-0.01 to 0.05) | .115 |  |  |  |  |  |  |  |  |  |  |
| **Total accumulated QALY (Spain)** | | | | | | | | | | | | | | | |
| Month 6 |  |  |  |  |  | 114/130 | 0.857 (0.02) | 0.784 (0.02) | 0.074 (0.02 to 0.13) | .006 | 124/137 | 1.04 (0.02) | 0.845 (0.02) | 0.194 (0.14 to 0.25) | <.001 |
| Month 12 |  |  |  |  |  | 88/98 | 0.775 (0.02) | 0.756 (0.02) | 0.018 (-0.04 to 0.08) | .541 | 100/113 | 0.763 (0.02) | 0.803 (0.02) | -0.040 (-0.10 to 0.02) | .181 |
| Month 18 |  |  |  |  |  | 63/64 | 0.808 (0.03) | 0.723 (0.02) | 0.085 (0.02 to 0.15) | .016 | 89/101 | 0.721 (0.02) | 0.743 (0.02) | -0.022 (-0.08 to 0.04) | .491 |
| Abbreviations: BTH: Blekinge Institute of Technology, Karlskrona, Sweden; CST: Consorci Sanitaria de Terrassa, Barcelona, Spain; QALY: quality adjusted life years; SAS: Service Andaluz de Salud, Málaga, Spain; ZBI: Zarit Caregiver Burden Inventory.  a: (Intervention group/Control group); b: Mean (Standard Error); c: P-values for exploratory outcomes are nominal and have not been adjusted for multiple comparisons. | | | | | | | | | | | | | | | |

| **Table S10**  *Change in medication adherence (≥ 80% and ≤ 110%) between intervention and control group at 6-month, 12-month, and 18-month follow-up by site* | | | | | | | | | | |
| --- | --- | --- | --- | --- | --- | --- | --- | --- | --- | --- |
|  | For both drugs | | | | | At least one drug | | | | |
|  | Sample size | Intervention group | Control group | Absolute difference in probabilities | | Sample size | Intervention group | Control group | Absolute difference in probabilities | |
|  | (I/C)^a^ | Mean (SE)^b^ | Mean (SE)^b^ | Difference (95% CI) | *P-value* ^c^ | (I/C)^a^ | Mean (SE)^b^ | Mean (SE)^b^ | Difference (95% CI) | *P-value* ^c^ |
| **BTH** | | | | | | | | | | |
| 6-month | 72/93 | 0.341 (0.06) | 0.410 (0.05) | -0.069 (-0.22 to 0.08) | .359 | 94/106 | 0.743 (0.04) | 0.820 (0.04) | -0.077 (-0.19 to 0.04) | .186 |
| 12-month | 66/86 | 0.342 (0.06) | 0.568 (0.05) | -0.226 (-0.38 to -0.07) | .004 | 83/103 | 0.747 (0.05) | 0.767 (0.04) | -0.019 (-0.14 to 0.10) | .758 |
| 18-month | 62/75 | 0.478 (0.06) | 0.496 (0.06) | -0.018 (-0.19 to 0.15) | .829 | 79/94 | 0.813 (0.04) | 0.780 (0.04) | 0.033 (-0.09 to 0.15) | .595 |
| **CST** | | | | | | | | | | |
| 6-month | - | - |  |  |  | 82/80 | 0.733 (0.05) | 0.608 (0.05) | 0.125 (-0.02 to 0.27) | .087 |
| 12-month | - | - |  |  |  | 53/27 | 0.678 (0.06) | 0.715 (0.08) | -0.038 (-0.24 to 0.17) | .721 |
| 18-month | - | - |  |  |  | 42/36 | 0.654 (0.07) | 0.670 (0.08) | -0.160 (-0.23 to 0.19) | .882 |
| **SAS** | | | | | | | | | | |
| 6-month | 83/90 | 0.470 (0.05) | 0.396 (0.05) | 0.074 (-0.07 to 0.22) | .325 | 109/113 | 0.798 (0.04) | 0.665 (0.04) | 0.133 (0.02 to 0.25) | .023 |
| 12-month | 39/69 | 0.505 (0.08) | 0.397 (0.06) | 0.108 (-0.09 to 0.30) | .275 | 65/80 | 0.786 (0.05) | 0.778 (0.05) | 0.008 (-0.13 to 0.14) | .907 |
| 18-month | 68/65 | 0.438 (0.06) | 0.332 (0.06) | 0.106 (-0.06 to 0.27) | .204 | 88/79 | 0.763 (0.05) | 0.745 (0.05) | 0.018 (-0.11 to 0.15) | 0.792 |
| Abbreviation: BTH: Blekinge Institute of Technology, Karlskrona, Sweden; CST: Consorci Sanitaria de Terrassa, Barcelona, Spain; SAS: Service Andaluz de Salud, Málaga, Spain.  a: (Intervention group/Control group); b: Mean (Standard Error); c: P-values for exploratory outcomes are nominal and have not been adjusted for multiple comparisons.  Note: Estimates for CST for “both drugs” could not be obtained due to non-convergence resulting from discontinuities in the likelihood function and missing values. | | | | | | | | | | |

| **Table S11**  *Differences in baseline characteristics of dropout and non-dropout PwMCI* | | | | | | |
| --- | --- | --- | --- | --- | --- | --- |
| Characteristics | Intervention Group | | | Control Group | | |
|  | Dropout (n=225) | Non-dropout (n=312) | *P* value | Dropout  (n=204) | Non-dropout  (n=337) | *P* value |
| Age (years)^a^ | 76 (7.52) | 74 (6.91) | <.001 | 75 (7.94) | 74 (6.76) | .478 |
| Sex, n (%) | | | | | | |
| Male | 85 (38) | 156 (50) | .005 | 92 (45) | 173 (51) | .160 |
| Female | 140 (62) | 156 (50) |  | 112 (55) | 164 (49) |  |
| Education, n (%) | | | | | | |
| Elementary education | 134 (60) | 179 (57) | .842 | 132 (65) | 201 (60) | .046 |
| Secondary education | 50 (22) | 74 (24) |  | 42 (21) | 58 (17) |  |
| Higher education | 39 (18) | 58 (19) |  | 29 (14) | 77 (23) |  |
| Marital Status, n (%) | | | | | | |
| Unmarried | 88 (39) | 93 (30) | .024 | 75 (37) | 84 (25) | .003 |
| Married/Partner | 137 (61) | 219 (70) |  | 128 (63) | 253 (75) |  |
| Living arrangements, n (%) | | | | | | |
| Living alone | 48 (22) | 69 (22) | .019 | 44 (22) | 58 (17) | .032 |
| Spouse/Common law partner | 125 (57) | 202 (65) |  | 118 (59) | 238 (70) |  |
| Children | 34 (15) | 22 (7) |  | 19 (9) | 22 (7) |  |
| Other | 13 (6) | 19 (6) |  | 20 (10) | 19 (6) |  |
| EQ-5D-3L index scores ^a, b^ (EVAS) | 0.76 (0.20) | 0.78 (0.19) | .148 | 0.75 (0.22) | 0.79 (0.20) | .071 |
| QoL-AD ^a, b^ | 35.848 (5.97) | 36.622 (6.42) | .157 | 34.78 (6.48) | 36.90 (6.69) | <.001 |
| MMSE score^a^ | 24.51 (2.76) | 26.02 (2.11) | <.001 | 24.88 (2.55) | 25.86 (2.23) | <.001 |
| IADL score^a,c^ | 6.22 (1.87) | 7.05 (1.40) | <.001 | 6.36 (1.71) | 6.83 (1.67) | .003 |
| Abbreviations: IADL: Instrumental activities of daily living; MMSE mini-mental state examination; PwMCI: persons with mild cognitive impairment; QoL-AD: quality of life in Alzheimer disease; n: number; % percentage  Note*:* a: Mean (standard deviation); b: n=224 for dropout in the intervention group; c: n=198 for dropout; n=273 for non-dropout in the intervention group and n=190 for dropout and n=297 for non-dropout in the control group.  Independent sample t-test is used to assess the statistical differences between dropouts and non-dropouts (inter-group [between groups] analysis). | | | | | | |

| **Table S12**  *Differences in baseline characteristics of dropout and non-dropout informal caregiver* | | | | | | |
| --- | --- | --- | --- | --- | --- | --- |
| Characteristics | Intervention Group | | | Control Group | | |
|  | Dropouts (n=225) | Non-dropouts (n=312) | *P* value | Dropouts  (n=204) | Non-dropouts (n=337) | *P* value |
| Age (years)^a^ | 60 (16.16) | 63 (13.94) | .006 | 61 (13.97) | 64 (14.54) | .068 |
| Sex, n (%) | | | | | | |
| Male | 79 (35) | 87 (28) | .074 | 67 (33) | 111 (33) | .982 |
| Female | 146 (65) | 225 (72) |  | 137 (67) | 226 (67) |  |
| Education, n (%) | | | | | | |
| Elementary education | 64 (29) | 111 (37) | .084 | 81 (41) | 120 (36) | .537 |
| Secondary education | 76 (35) | 110 (36) |  | 54 (27) | 102 (31) |  |
| Higher education | 78 (36) | 83 (27) |  | 64 (32) | 109 (33) |  |
| Marital Status, n (%) | | | | | | |
| Unmarried | 49 (22) | 56 (18) | .248 | 38 (19) | 57 (17) | .594 |
| Married/Partner | 174 (78) | 256 (82) |  | 165 (81) | 280 (83) |  |
| Living arrangements, n (%) | | | | | | |
| Living alone | 18 (8) | 34 (11) | .009 | 17 (8) | 26 (8) | .199 |
| Spouse/Common law partner | 137 (62) | 220 (70) |  | 133 (65) | 245 (73) |  |
| Children | 25 (11) | 28 (9) |  | 24 (12) | 24 (7) |  |
| Other | 42 (19) | 30 (10) |  | 30 (15) | 42 (12) |  |
| EQ-5D-3L index scores ^a, c^ (EVAS) | 0.788 (0.19) | 0.810 (0.18) | .169 | 0.790 (0.21) | 0.783 (0.21) | .734 |
| QoL-AD ^a, b^ | 33.06 (6.76) | 35.32 (7.31) | <.001 | 33.41 (6.59) | 35.14 (7.10) | .005 |
| ZBI^a, d^ | 7.76 (7.43) | 6.04 (7.37) | .008 | 8.09 (8.53) | 6.12 (7.38) | .005 |
| Abbreviations: QoL-AD: quality of life in Alzheimer disease; ZBI: Zarit Caregiver Burden Inventory; n number; % percentage  Note: a: Mean (standard deviation); b: n= 223 for dropout in intervention group & n= 203 for dropout in control group; c: n= 224 for dropout in intervention group & n= 202 for dropout in control group; d: n= 224 for dropout in intervention group & n= 203 for dropout in control group; Independent sample t-test is used to assess the statistical differences between dropouts and non-dropouts (inter-group [between groups] analysis). | | | | | | |

| **Table S13**  **Tipping point analyses applying δ shifts** | | | |
| --- | --- | --- | --- |
| **Delta (δ)** | **Mean difference at 18-month** | **95 % CI** | ***P*-value** |
| **PwMCI** |  |  |  |
| **Composite QoL-AD** |  |  |  |
| 0 (MAR) | 0.731 | 0.12 to 1.34 | .018 |
| -4 | 0.842 | 0.22 to 1.46 | .008 |
| -2 | 0.787 | 0.18 to 1.40 | .011 |
| 2 | 0.674 | 0.06 to 1.29 | .031 |
| 4 | 0.617 | -0.01 to 1.24 | .052 |
| **Imputed QALY** |  |  |  |
| 0 (MAR) | 0.010 | -0.003 to 0.02 | .127 |
| -0.07 | 0.007 | -0.006 to 0.02 | .312 |
| -0.05 | 0.007 | -0.005 to 0.02 | .246 |
| -0.02 | 0.009 | -0.004 to 0.02 | .167 |
| 0.02 | 0.011 | -0.002 to 0.02 | .096 |
| 0.05 | 0.012 | -0.001 to 0.02 | .063 |
| 0.07 | 0.013 | 0.0002 to 0.03 | .047 |
| **Informal Carer** |  |  |  |
| **Imputed QALY** |  |  |  |
| 0 (MAR) | 0.014 | 0.003 to 0.03 | .016 |
| -0.15 | 0.012 | -0.001 to 0.02 | .082 |
| -0.10 | 0.013 | 0.0002 to 0.02 | .047 |
| -0.09 | 0.013 | 0.0005 to 0.03 | .042 |
| 0.09 | 0.016 | 0.004 to 0.03 | .009 |
| 0.10 | 0.016 | 0.004 to 0.03 | .008 |
| 0.15 | 0.017 | 0.005 to 0.03 | .008 |

| **Table S14**  *Percentage of missing/ imputed values for imputed outcome measures* | | | | | | |
| --- | --- | --- | --- | --- | --- | --- |
|  | PwMCI | | | | Informal Carer | |
|  | QoL-AD | | QALY | | QALY | |
|  | Sample size | % Missing/ imputed* | Sample size | % Missing/ imputed | Sample size | % Missing/ imputed |
| Total values at baseline | 1078 |  | 1078 |  | 1078 |  |
| Missing at M6 | 243 | 22.5 | 239 | 22.2 | 243 | 22.5 |
| Imputed at M6 | 233 | 21.6 | 232 | 21.5 | 234 | 21.7 |
| Missing at M12 | 501 | 46.5 | 330 | 30.6 | 348 | 32.3 |
| Imputed at M12 | 280 | 26.0 | 321 | 30.0 | 337 | 31.3 |
| Missing at M18 | 449 | 41.7 | 433 | 40.2 | 436 | 40.4 |
| Imputed at M18 | 136 | 12.6 | 420 | 39.0 | 420 | 40.0 |
| *All missing data was not imputed due to missing data for some of the outcome variables (education, living arrangement, QoL-AD, and EQ-5D-3L index scores (EVAS)) at baseline. | | | | | | |

**References**

[1]	Lavori PW, Dawson R, Shera D (1995) A multiple imputation strategy for clinical trials with truncation of patient data. *Stat Med* **14**, 1913-1925.

[2]	Rubin D (1987) *Multiple Imputation for Nonresponse in Surveys*, Wiley & Sons, New York.
